# POSTN‐Mediated Interplay of M1 Polarized Macrophage with Tendon‐Derived Stem Cells to Drive Traumatic Heterotopic Ossification Formation through PTK7/ATK Signaling?

**DOI:** 10.1002/advs.202507951

**Published:** 2025-08-18

**Authors:** Hang Liu, Xinyue Li, Mengyi Li, Ziyang Sun, Xu Wang, Juehong Li, Binbin Xu, Qian Chen, Cunyi Fan, Hongjiang Ruan

**Affiliations:** ^1^ Department of Orthopedics Shanghai Sixth People's Hospital Affiliated to Shanghai Jiao Tong University School of Medicine Shanghai 200233 P. R. China; ^2^ Shanghai Engineering Research Center for Orthopaedic Material Innovation and Tissue Regeneration Shanghai 201306 P. R. China; ^3^ Shanghai General Hospital Shanghai 200000 P. R. China; ^4^ Shanghai Pudong New Area GongliHospital Shanghai 200135 China

**Keywords:** heterotopic ossification, macrophage polarization, osteogenic differentiation, POSTN, secretions, TDSCs (tendon‐derived stem cells)

## Abstract

Tendinous heterotopic ossification can cause pain and restricted joint mobility in affected areas, and it is a common and severe complication following tendon injuries. This condition significantly reduces the postoperative quality of life of patients, and its incidence is increasing year by year. Due to the unclear pathogenesis, there are currently no effective treatment methods. Although recent studies suggest that macrophages affect the process of traumatic heterotopic ossification (HO) in mice, their role in HO still requires further clarification. Here, it is disclosed that the formation of trauma‐induced HO is accompanied by the polarization of macrophages toward the M1 phenotype. Additionally, secretion containing periostin (POSTN) that is secreted by M1 macrophages reduces fatty acid β – oxidation in tendon‐derived stem cells (TDSCs) and facilitates the formation of heterotopic bone. Mechanistically, M1 macrophages release POSTN during the HO process, which directly binds to PTK7 in TDSCs,  thereby increasing AKT phosphorylation at the S124 site and initiating osteogenic differentiation. This study demonstrates the role of M1 macrophages and their secreted POSTN in traumatic heterotopic ossification, highlighting the potential of POSTN as a therapeutic target for HO.

## Graphical abstract

The mechanism diagram reveals that the formation of trauma‐induced heterotopic ossification (HO) is accompanied by the polarization of macrophages toward the M1 phenotype. Additionally, the extracellular vesicles secreted by M1 macrophages, which contain periostin (POSTN), directly bind to PTK7 in tendon‐derived stem cells (TDSCs). This binding increases the phosphorylation of AKT at the S124 site, thereby reducing fatty‐acid β‐oxidation in TDSCs and promoting the formation of heterotopic bone.

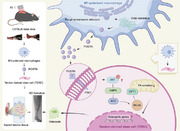

## 1 Introduction

Tendons are the connective tissues that transmit muscle activity to bones, facilitating joint movement.^[^ ^1^ ^]^ They play a crucial role in maintaining normal body load and movement posture. Unreasonable exercise intensity and improper posture are common causes of tendon injuries, and unhealthy lifestyle habits, such as prolonged sitting, also predispose individuals to tendon injuries in daily life.^[^ ^2^, ^3^ ^]^ Consequently, tendon injuries have become a common and frequently occurring condition in clinical practice. Post injury, tendons are highly susceptible to rupture, leading to impaired motor function and reduced quality of life in patients.^[^ ^4^ ^]^ Clinically, tendon injuries usually undergo surgical repair. However, long‐term complications like tendon calcification or ossification, which is referred to as tendinous heterotopic ossification (HO), often take place after the surgery. This condition is considered a form of traumatic HO.^[^ ^5^ ^]^ Tendinous HO is the result of abnormal repair following tendon injury and is a common severe complication. At present, there are no effective preventive or curative approaches, imposing a substantial burden on both patients and healthcare providers.^[^ ^6^ ^]^ Therefore, it is of great significance to explore the pathogenesis of tendinous HO as well as potential preventive and treatment measures.

Tendon‐derived stem cells (TDSCs) are a population of stem cells found in tendons that have the potential to differentiate into various cell types, including tenocytes and osteoblasts.^[^ ^7^ ^]^ Under normal conditions, TDSCs maintain tendon homeostasis by differentiating into tenocytes and participating in tendon repair and regeneration. However, in certain pathological conditions, such as after trauma or inflammation, TDSCs can be induced to differentiate into osteoblasts, leading to the formation of heterotopic bone.^[^ ^8^ ^]^ In recent years, numerous studies have indicated that the inflammatory microenvironment following tendon injury affected the fate of TDSCs, with spatial dialogue occurring between immune cells and TDSCs, among which macrophages play a crucial role in the immune response during HO.^[^ ^9^, ^10^, ^11^ ^]^

Macrophages can be polarized into M1 or M2 phenotypes in response to different stimulation.^[^ ^12^ ^]^ Recent studies have suggested that macrophage polarization can influence the osteogenic differentiation of TDSCs.^[^ ^13^ ^]^ M1 macrophages have been shown to promote the osteogenic differentiation of TDSCs through the secretion of pro‐inflammatory cytokines such as tumor necrosis factor‐α (TNF‐α) and interleukin‐1β (IL‐1β).^[^ ^14^, ^15^ ^]^ These cytokines can activate signaling pathways such as the NF‐κB pathway and the MAPK pathway in TDSCs, leading to the upregulation of osteogenic transcription factors and the promotion of osteoblast differentiation.^[^ ^16^ ^]^ The polarization direction of macrophages can influence cell fate, with M1 polarization inducing osteogenic‐biased differentiation of TDSCs.^[^ ^17^ ^]^ Although recent studies have provided important insights into the relationship between macrophage polarization and the osteogenic differentiation of TDSCs. The specific molecular mechanisms by which macrophage polarization regulates the osteogenic differentiation of TDSCs are still not fully understood.

Paracrine is an important mediator of intercellular communication, secreting a large amount of molecular information such as DNA, RNA, and proteins.^[^ ^18^ ^]^ Proteins secreted by macrophages in obese mice have been shown to cause shifts in the differentiation of skeletal stem/progenitor cells between osteoblasts and adipocytes, as well as skeletal degeneration.^[^ ^19^ ^]^ Macrophage‐derived proteins have been shown to alleviate the senescence of mesenchymal stem cells in the bone marrow by regulating inflammatory responses, enhancing chondrogenesis, and promoting tendon‐bone healing.^[^ ^20^ ^]^ Moreover, researchers have found that oncostatin M secreted by macrophages can promote the expression of runt‐related transcription factor 2 (Runx2) within bone marrow‐derived stem cells, thereby facilitating their osteogenic differentiation.^[^ ^21^ ^]^ These studies collectively highlight the capacity of macrophage‐secreted proteins to regulate the differentiation direction and osteogenic commitment of mesenchymal stem cells. Energy metabolism also plays a regulatory role in the differentiation process of stem cells, with cellular metabolites and their derivatives being able to alter gene expression related to differentiation. Recent studies have identified that initiating a metabolic shift from fatty acid oxidation (FAO) to glycolysis promotes osteoclast differentiation in osteoporosis.^[^ ^22^ ^]^ This metabolic reprogramming in stem cells profoundly affects cell differentiation fate. However, the role and mechanism of macrophage‐derived proteins and metabolic reprogramming in TDSCs during HO induced by Achilles tendon injury have not been fully elucidated.

In this study, we investigated the influence of macrophages on the abnormal osteogenesis of TDSCs and the mechanisms of cellular dialogue between macrophages and TDSCs during the formation of HO following tendon injury. Our findings provide new mechanistic insights into how inflammatory macrophages induce HO formation in the injured tendon and suggest new directions for future disease prevention and intervention.

## 2 Results

### 2.1 Increased Polarization of M1 Macrophages in Trauma‐Induced Heterotopic Ossification

**Figure 1:**
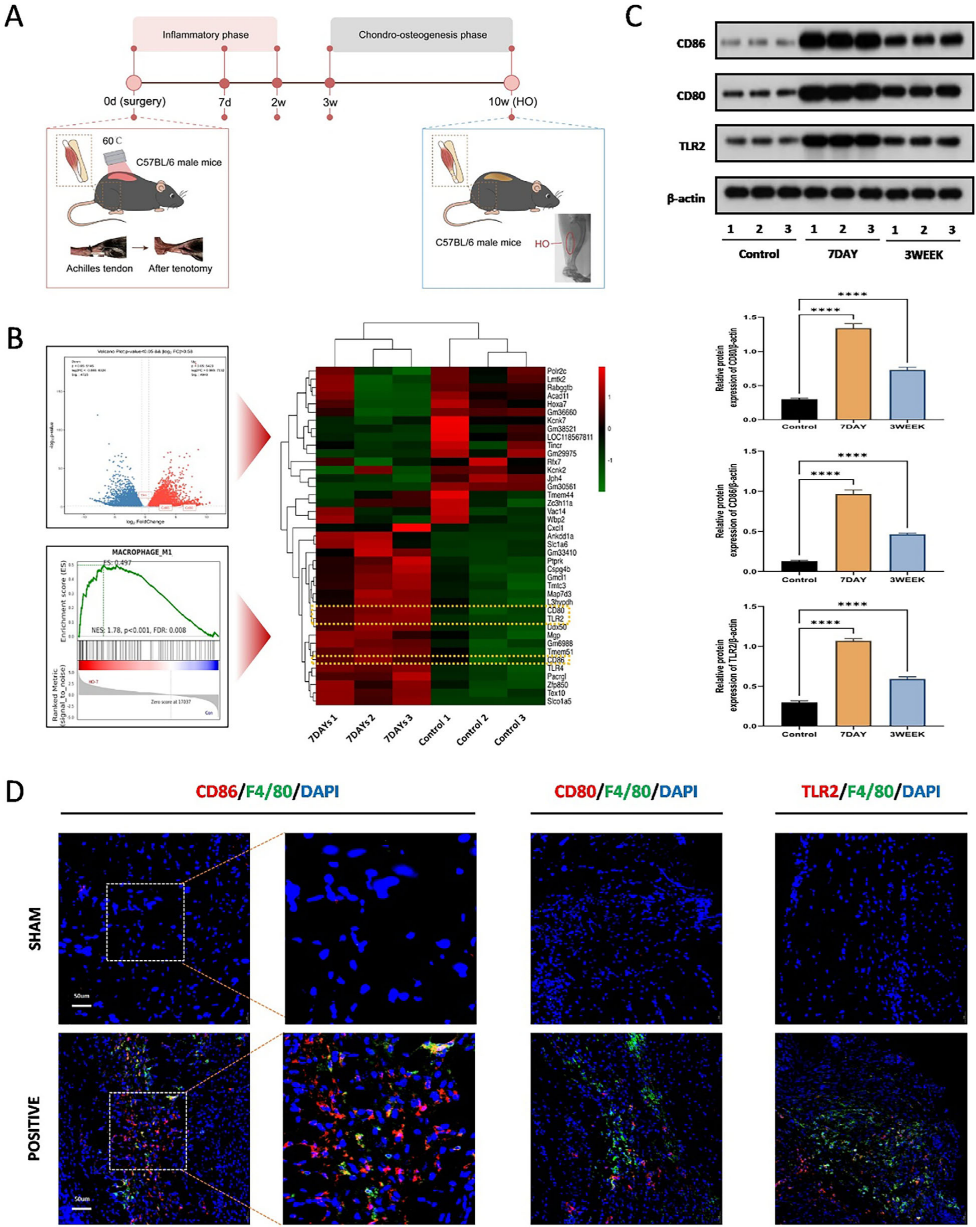
Increased polarization of M1 macrophages in trauma‐induced heterotopic ossification. A) Schematic depiction of the murine traumatic HO model and its critical developmental stages. B) Gene set enrichment analysis of macrophage M1‐associated gene sets, with corresponding volcano plot and heatmap illustrating differential gene expression in Control and HO groups at 7 days, *N* = 3. C) Western blot analysis of macrophage M1‐associated protein dynamics, including CD86, CD80, and TLR2, via WB at distinct stages of HO formation, *N* = 3, **** *p* < 0.0001. D) Double immunofluorescence staining for the co‐localization of CD86, CD80, TLR2and F4/80 positive cells in both Sham and HO groups at 7 days, *N *= 3, scale bar = 200 µm.

In order to determine the changes during the formation of trauma‐induced HO, we constructed a mouse burn/tendon injury model that simulates the formation of trauma‐induced HO (**Figure** **1**A). Heterotopic ossification tissues collected after 7 days injury were subjected to transcriptome sequencing analysis, which revealed that the expression of genes related to M1 macrophages was upregulated in HO tissues, leading to high enrichment of M1 macrophage polarization marker genes in GSEA analysis (Figure 1B). To further validate the bioinformatics findings and for more detailed exploration, the polarization process of M1 macrophages was characterized at different time points during the evolution of HO in the mouse model by WB analysis. The results showed that the protein levels of molecules related to M1 macrophage polarization, including CD80, CD86, and TLR2, were significantly enhanced in the early stages of trauma‐induced HO. Furthermore, we found that the level of M1 macrophage polarization peaked 7 days after injury (Figure 1C; Figure S1A,B, Supporting Information). In addition, immunofluorescence staining showed that macrophage marker F4/80 and M1 macrophage polarization markers CD80, CD86, and TLR2 all significantly increased, and co‐localization was observed (Figure 1D). Similarly, the M1 polarization of macrophages was also detected and confirmed at 3 weeks after surgery (Figure S1A,B, Supporting Information). This indicates that there is an induction of macrophage polarization toward the M1 phenotype during HO formation, which is consistent with the results of WB.

### 2.2 Knocking out NF‐κB Inhibits Macrophage Polarization toward the M1 Phenotype and Reduces Heterotopic Ossification Formation after Trauma

**Figure 2:**
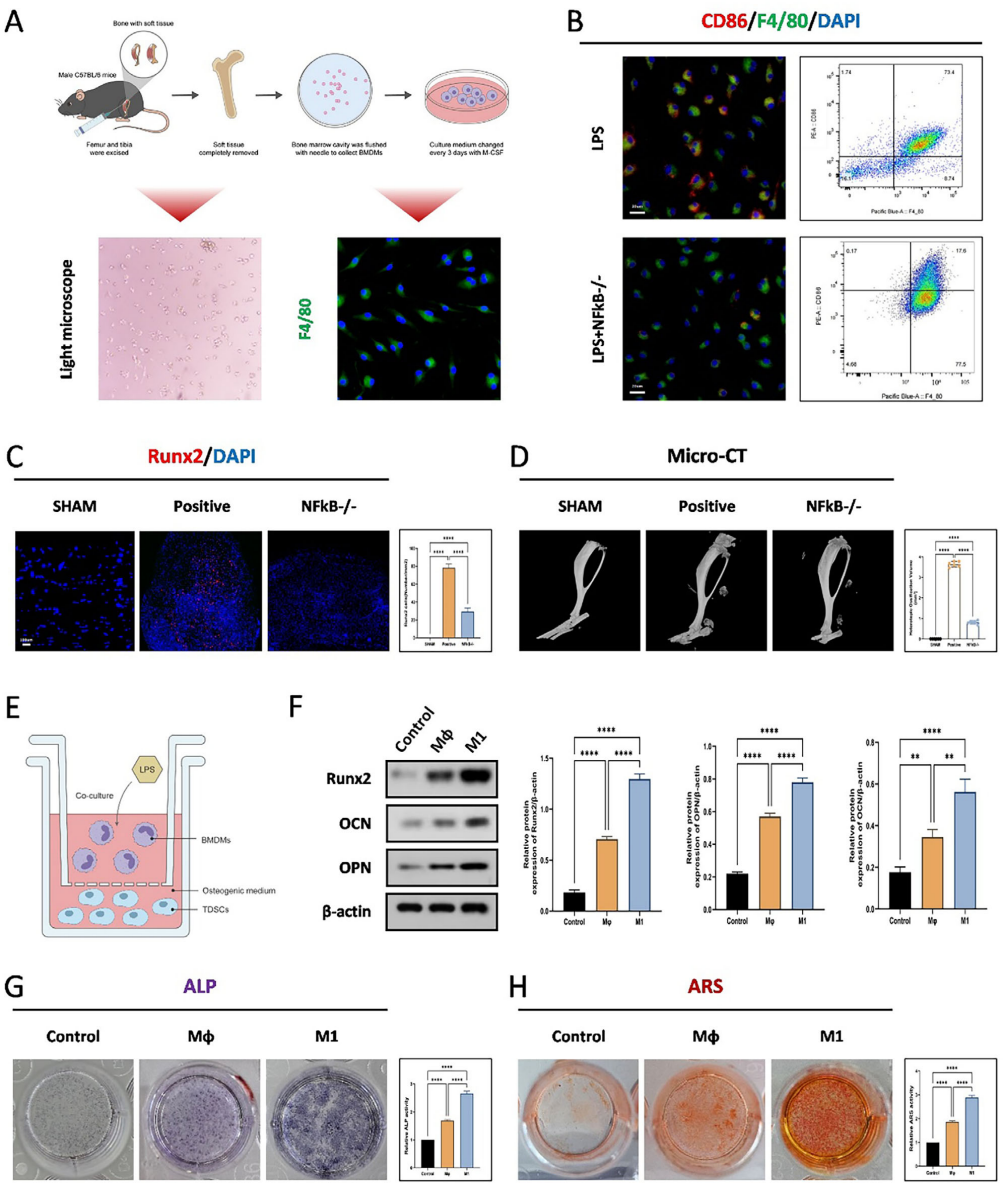
Knocking out NF‐κB inhibits macrophage polarization toward the M1 phenotype and reduces heterotopic ossification formation after trauma. A) Schematic illustrating the extraction of BMDMs from bone marrow and their differentiation into M1 macrophages induced by M‐CSF. B) Immunofluorescence staining and Flow Cytometry for the CD86 and F4/80 in both LPS and LPS with NFκB knockout groups, *N* = 3. C) Immunofluorescence staining for the Runx2 and PDGFRα in the sham, positive, and NFκB knockout groups, *N* = 6, ****p < 0.0001. D) Micro‐CT analysis of HO formation in the sham, positive, and NFκB knockout groups at 10 weeks post‐burn/tenotomy, *N* = 6, ****p < 0.0001. E) Illustration of the co‐culture system with LPS‐induced BMDMs and TDSCs with osteogenic induction. F) Western blot analysis of osteogenic‐related protein levels of TDSCs (Runx2, OCN, OPN) following indicated treatments at 7 days after osteogenic induction, *N* = 3, **p < 0.01, ****p < 0.0001. G) ALP staining for TDSCs in the indicated groups, *N* = 6, ****p < 0.0001. H) ARS staining for TDSCs in the indicated treatments, *N* = 6, ****p < 0.0001.

Previous studies have shown that the NF‐κB signaling pathway can mediate the polarization of M1‐type macrophages. To elucidate the effect of macrophage polarization on HO formation after injury, we isolated bone marrow‐derived macrophages from wild‐type (WT) mice and global NF‐κB gene knockout (NF‐κB −/−) mice for in vitro culture and induced them to become M1 macrophages (**Figure** **2**A). After burn/tendon injury, immunofluorescence staining and flow cytometry experiment results showed that the formation of M1‐type macrophage polarization was reduced in NF‐κB−/− mice compared to wild‐type mice (Figure 2B). Immunofluorescence staining for Runx2 and Micro‐CT measurements revealed a significant reduction in HO formation after NF‐κB knockout (Figure 2C,D). Furthermore, Safranine O staining and H&E staining showed that M1 macrophages have osteogenic effects, and the knockout of NF‐κB could reverse this effect (Figure S2A,B, Supporting Information). Given that the effects of macrophages are mainly achieved through their paracrine functions, we explored whether the secretions of M1 macrophages were responsible for the osteogenic effect on TDSCs. A co‐culture of macrophages and TDSCs was established using a transwell system, and macrophages were induced to polarize toward the M1 phenotype with LPS stimulation (Figure 2E). WB results indicated that the secretions from M1 macrophages, compared to M0 macrophages, could significantly enhance the expression of osteogenic‐related proteins runt‐related transcription factor 2 (Runx2), osteocalcin (OCN), and osteopontin (OPN) in TDSCs (Figure 2F). Immunofluorescence staining results also indicated that M1 macrophages could promote the expression of osteogenic‐related proteins Runx2, OCN, and OPN (Figure S2C, Supporting Information). At the same time, ALP and alizarin red S ARS staining showed that M1 macrophages could induce an enhanced osteogenic effect (Figure 2G,H). These results suggest that M1 macrophages are involved in the formation of HO.

### 2.3 Soluble Factors Secreted by M1 Macrophages Promote Trauma‐Induced HO Formation

**Figure 3:**
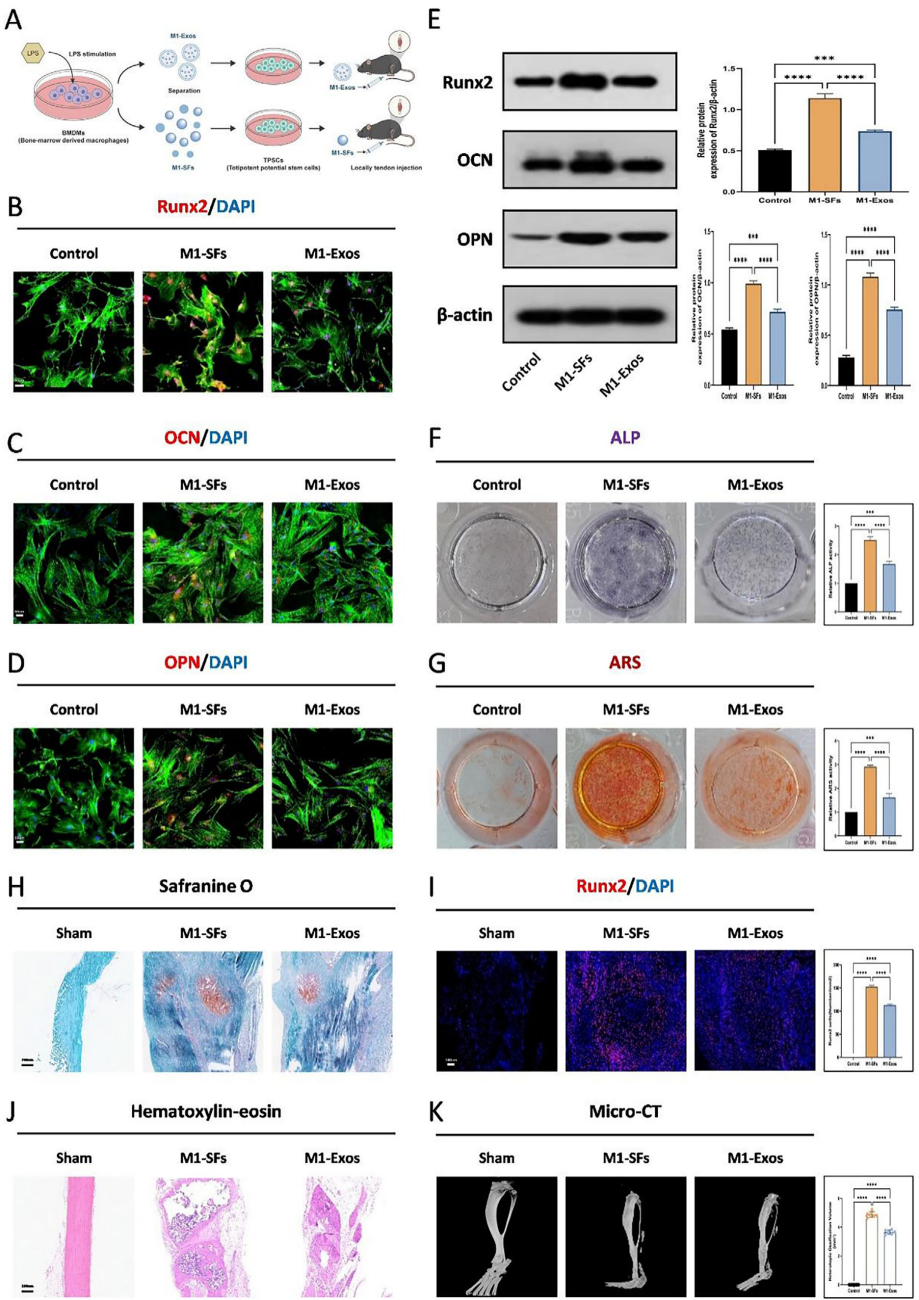
Soluble components secreted by M1 macrophages promote trauma‐induced HO formation. A) Schematic diagram of the experimental procedure involving TDSCs treated with distinct M1 supernatant fractions (M1‐SFs, M1‐Exos). B–D) Immunofluorescence staining for the Runx2 (B), OCN (C), OPN (D) in the Control, M1‐SFs, and M1‐Exos groups, *N* = 3. E) WB analysis was used to detect the expression of osteogenic‐related protein levels (Runx2, OCN, OPN) in the osteogenic induced TDSCs in addition of control, M1‐SFs, or M1‐Exos, *N* = 3, ***p < 0.001, ****p < 0.0001. F) ALP staining for TDSCs in the control, M1‐SFs, and M1‐Exos groups, *N* = 6, ****p < 0.0001. G) ARS staining for TDSCs in the control, M1‐SFs, and M1‐Exos groups, *N* = 6, ****p < 0.0001. H) Safranine O staining for tendon in the sham, M1‐SFs, and M1‐Exos groups, *N* = 6. I) Runx2 immunofluorescence staining in the sham, M1‐SFs and M1‐Exos groups, *N* = 6, ****p < 0.0001. J) H&E staining for tendon in the sham, M1‐Fs, and M1‐Exos groups, *N* = 6. K) Micro‐CT analysis of HO formation in the sham, M1‐SFs, and M1‐Exos groups at 10 weeks post‐burn/tenotomy, *N* = 6, ****p < 0.0001.

Since the secretions of M1 macrophages can be mainly divided into exosomes (Exos) and soluble factors (SFs), which conditioned medium without EVs. To assess the role of M1 macrophage secretions in trauma‐induced HO formation, we isolated Exos and SFs from the supernatant of M1 macrophage culture medium. Characterization of the isolated Exos was carried out by means of transmission electron microscopy (TEM), nanoparticle tracking analysis, and Western blot (WB) analysis. Two components were used respectively to treat TDSCs and to be injected into the tendons of mice (**Figure** **3**A; Figure S3A–D, Supporting Information). To verify whether Exos or SFs play a role in osteogenic formation, we found through immunofluorescence staining and WB that SFs secreted by M1 macrophages, compared to Exos, could significantly induce the expression of osteogenic‐related proteins Runx2, OCN, and OPN in TDSCs (Figure 3B–E). Similarly, ALP and ARS staining also confirmed that SFs secreted by M1 macrophages enhanced the osteogenic effect, while the osteogenic effect of Exos was not as strong as that of SFs (Figure 3F,G). In animal models, Safranine O staining and H&E staining showed that the osteogenic effect induced by SFs secreted by M1 macrophages was more than that of Exos (Figure 3H,J). Furthermore, in terms of Runx2 immunofluorescence staining and Micro‐CT measurements, SFs also had a stronger promoting effect on bone formation than Exos (Figure 3I,K).

### 2.4 POSTN in M1 Macrophage‐Derived SFs Mediates the Formation of Traumatic HO

**Figure 4:**
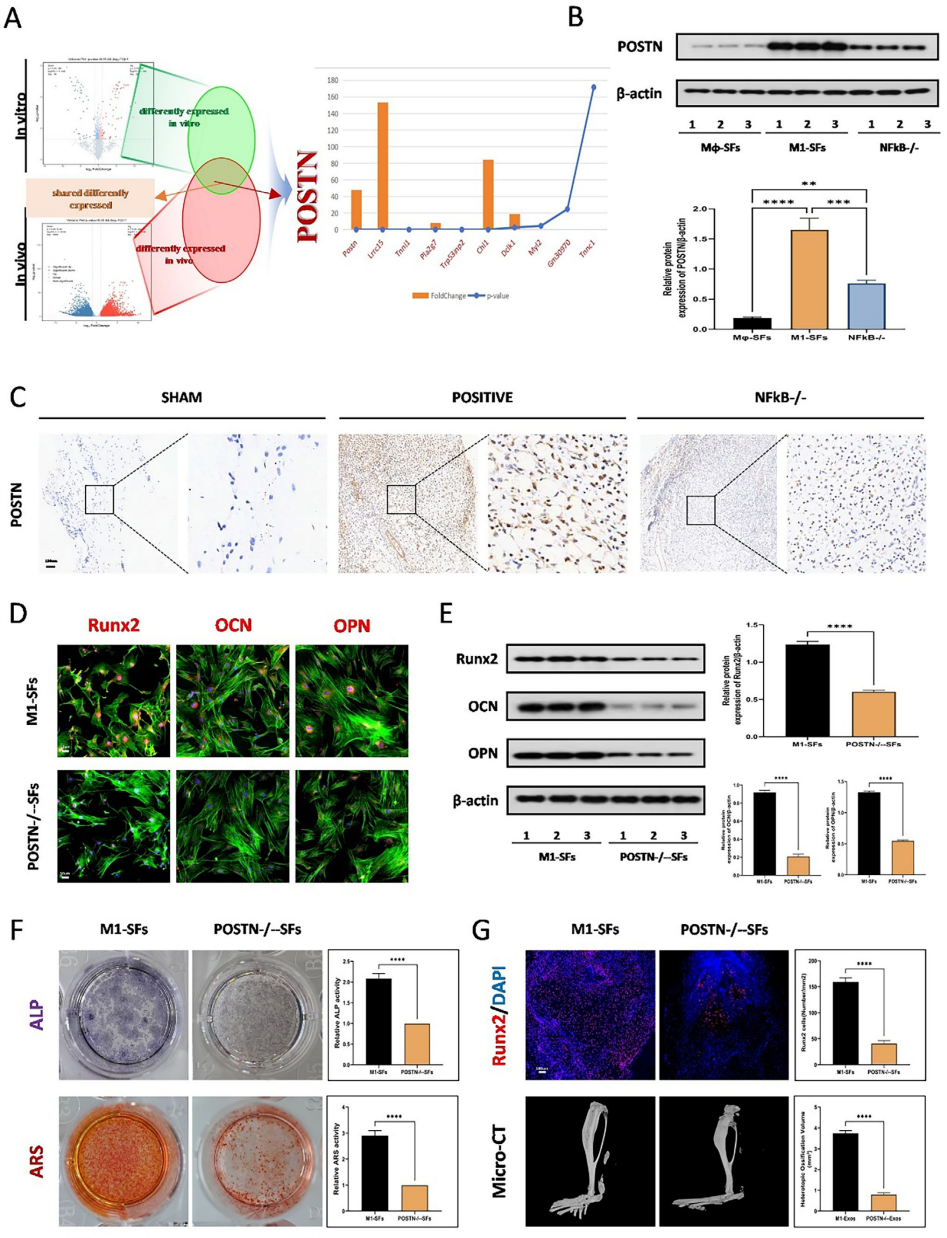
POSTN in M1 macrophage‐derived SFs mediates the formation of traumatic HO. A) High‐throughput sequencing was performed between SFs derived from macrophages and M1‐macrophages in vitro and between the sham group and the tendon lesions at 7 days in vivo. An intersection Venn diagram was drawn. B) WB analysis was used to detect the expression of POSTN proteins in Mφ‐SFs, M1‐SFs, and NFκB knock out groups, *N* = 3, **p < 0.01, ***p < 0.001, ****p < 0.0001. C) IHC staining was used to detect the expression of POSTN in the sham, positive, and NFκB knock‐out groups, *N* = 6. D) Immunofluorescence staining for the Runx2, OCN, OPN for TDSCs in the M1‐SFs and M1‐SFs with POSTN knockout groups, *N* = 3. E) WB analysis was used to detect the expression of osteogenic‐related protein levels (Runx2, OCN, OPN) for TDSCs in the M1‐SFs and M1‐SFs with POSTN knockout groups, *N* = 3, ****p < 0.0001. F) ALP and ARS staining for TDSCs in the M1‐SFs and M1‐SFs with POSTN knockout groups, *N* = 6, ****p < 0.0001. G) Immunofluorescence staining for the Runx2 of tendons and Micro‐CT analysis of HO formation in the M1‐SFs and M1‐SFs with POSTN knockout groups, *N* = 6, ****p < 0.0001.

To further elucidate how M1 macrophages mediate the formation of trauma‐induced HO through SFs, we performed high‐throughput sequencing on the SFs derived from M1 macrophages and the mouse HO tissues respectively. We listed the differentially expressed genes in detail. The results showed that the POSTN protein was significantly highly expressed both in the HO tissues and in the supernatant secreted by M1 macrophages, and it ranked first (**Figure** **4**A). Therefore, we selected the POSTN protein for subsequent research. WB and Immunohistochemistry results collectively indicated that treatment with SFs significantly promoted the expression of POSTN protein, and this effect was reversed after knocking out NF‐κB in M1 macrophages (Figure 4B,C). Furthermore, we used tissue‐specific co‐staining and found that the POSTN protein had a very high degree of co‐localization with the M1 macrophage marker CD86. However, the co‐localization between the POSTN protein and the classical tendon stem cell markers PDGFRα, MKX, and SCX was relatively low (Figure S4A, Supporting Information). This indicates that the POSTN protein is mainly secreted by M1 macrophages. Furthermore, we generated POSTN knockout mice to further investigate the effect of POSTN protein on HO (Figure S4B, Supporting Information). To determine whether the POSTN protein itself influences tendon homeostasis, we performed comprehensive analyses using the following methods: macroscopic observations of tendon morphology, histological analysis (H&E and Masson's trichrome staining), and ultrastructural evaluation of collagen architecture via TEM. The results showed no significant differences in tendon homeostasis between knockout mice and wild‐type mice (Figure S4C, Supporting Information). We further verified the role of POSTN by constructing POSTN knockout mice and comparing the osteogenic effects of SFs secreted by M1 macrophages with or without POSTN and through safranin and HE staining, it was found that the HO area significantly decreased after POSTN knockout (Figure S4D,E, Supporting Information). Immunofluorescence staining and WB results showed that SFs lacking POSTN protein could not promote the expression of osteogenic‐related proteins Runx2, OCN, and OPN (Figure 4D,E). Similarly, ALP and ARS staining also confirmed that SFs lacking POSTN protein could reverse the osteogenic effect induced by SFs secreted by M1 macrophages (Figure 4F). In vivo, Runx2 immunofluorescence staining and Micro‐CT measurements also proved that the absence of POSTN could not mediate the osteogenic effect (Figure 4G). At the same time, Safranine O staining and H&E staining showed that SFs lacking POSTN protein did not have the effect of inducing osteogenesis (Figure S4F,G, Supporting Information). In order to further explore the role of macrophage‐derived POSTN in HO, after depleting macrophages using clodronate, WB analysis showed the amount of POSTN protein decreased significantly, approaching the level of POSTN knockout mice. This indicates that the POSTN protein secreted by macrophages accounts for the vast majority in HO tissues (Figure S4H, Supporting Information). After depleting macrophages using clodronate and then establishing an HO model, it was found that there was no significant difference in the level of HO between ordinary mice and POSTN knockout mice (Figure S4I, Supporting Information). This indicates that the POSTN protein derived from non‐macrophage sources does not significantly promote the formation of HO. In addition, micro‐CT and RUNX2 fluorescence staining showed that the in vivo use of recombinant periostin (rPOSTN) could reverse the effect of clodronate and there is no significant difference in the formation of HO when compared with the group treated with the supernatant of M1 macrophages, also suggesting that macrophage – derived POSTN protein plays a major role in the occurrence and development of HO and osteogenic differentiation of TDSCs (Figure S4J,K, Supporting Information). In in – vitro experiments, ALP and ARS staining reveal that the use of rPOSTN protein could significantly promote the osteogenic differentiation of TDSCs. However, the effect of using the POSTN protein inhibitor alone was not significant. This further indicates that exogenous POSTN protein plays a major role in the osteogenic differentiation of TDSCs (Figure S4L,M, Supporting Information).

### 2.5 POSTN Promotes the Formation of Traumatic HO by Inhibiting β‐Oxidation of Fatty Acids

**Figure 5:**
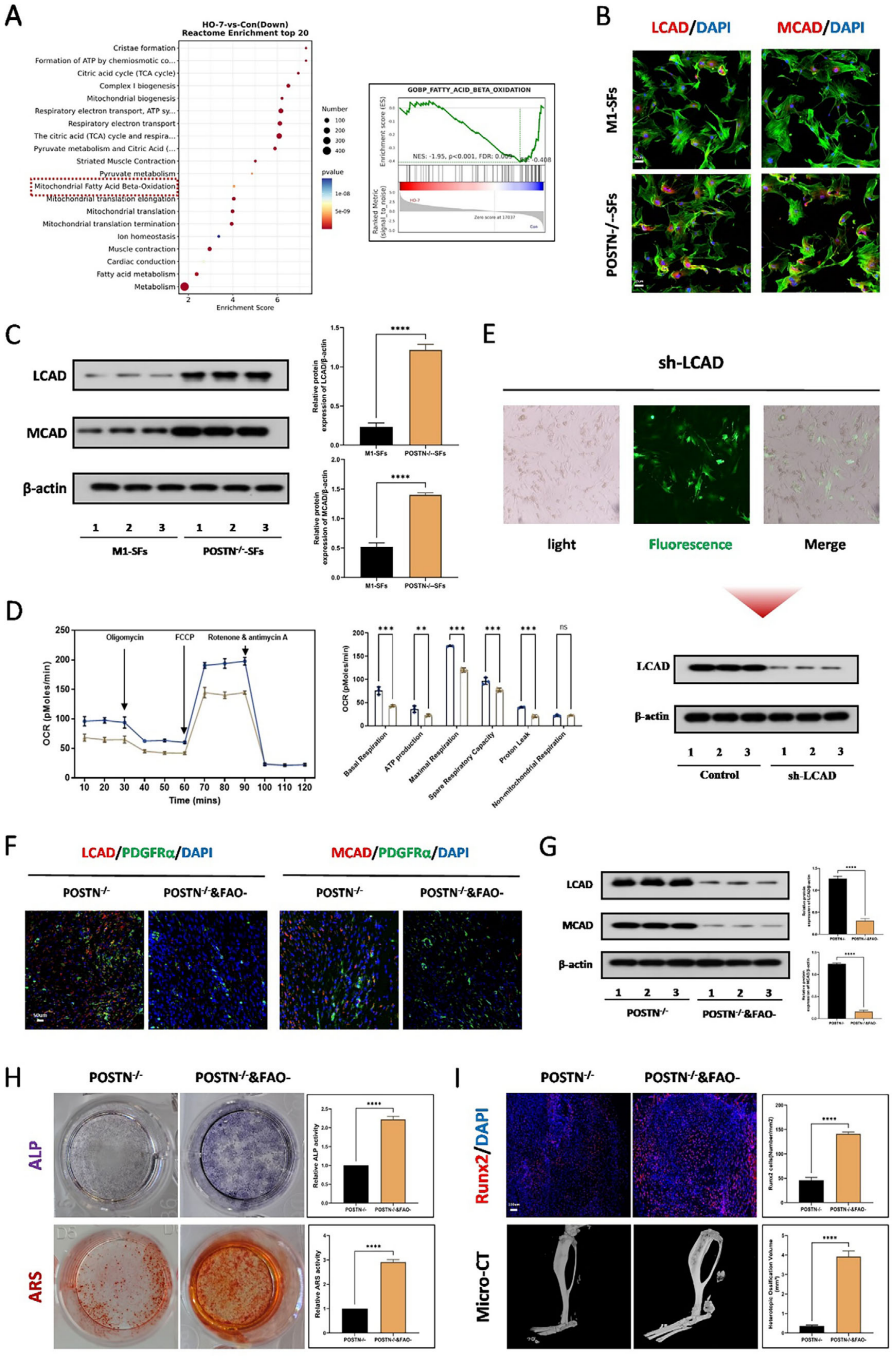
POSTN promotes the formation of traumatic HO by enhancing β‐oxidation of fatty acids. A) High‐throughput whole‐transcriptome sequencing was performed and showed by Reactome pathways enrichment analysis of RNA‐seq data between the sham group and tendon lesions at 7 days, *N* = 3 B) Immunofluorescence staining for LCAD and MCAD of TDSCs in the M1‐SFs and M1‐SFs with POSTN knockout groups. C) WB analysis was used to detect the expression of LCAD and MCAD proteins for TDSCs in the M1‐SFs and M1‐SFs with POSTN knockout groups, *N* = 3, ****p < 0.0001. D) Seahorse test was used to detect the oxidative phosphorylation level in the osteogenic induced TDSCs in addition of M1‐SFs or M1‐SFs with POSTN knockout groups, *N* = 3, ● represented M1‐SFs with POSTN knockout groups and ▲ represented M1‐SFs groups. E) Fluorescence and light microscope and WB analysis were used to confirm the success of downregulation of LCAD transfection for TDSCs, *N* = 3. F) Immunofluorescence staining for the LCAD and MCAD (red), co‐localized with PDGFRα(green) of tendons in addition of M1‐SFs with POSTN knockout groups, with or without sh‐LCAD, *N* = 3. G) WB analysis was used to detect the expression of LCAD and MCAD of TDSCs in addition of M1‐SFs with POSTN knockout groups, with or without sh‐LCAD, *N *= 3, ****p < 0.0001. H) ALP and ARS staining were used to detect the osteogenesis of TDSCs in addition of M1‐SFs with POSTN knockout groups, with or without sh‐LCAD, *N* = 6, ****p < 0.0001. I) Immunofluorescence staining for the Runx2 of tendons and micro‐CT analysis of HO formation in the M1‐SFs and M1‐SFs with POSTN knockout groups, *N* = 6, ****p < 0.0001.

To further clarify the mechanism by which POSTN mediates the formation of trauma‐induced HO, we performed Reactome metabolic pathway enrichment analysis and GSEA pathway analysis on downregulated differential genes, and the results showed significant enrichment of pathways related to mitochondrial fatty acid β‐oxidation (**Figure** **5**A). Immunofluorescence staining and WB showed that the absence of POSTN could reverse the significant downregulation of proteins related to fatty acid β‐oxidation, LCAD and MCAD, after M1 macrophage SFs treatment (Figure 5B,C). Subsequently, we conducted Seahorse energy metabolism analysis, and the results found that knocking down POSTN could significantly promote the mitochondrial oxidative respiratory capacity of TDSCs (Figure 5D). In order to further explore the role of FAO in the osteogenic differentiation of TDSCs, we transfected lentivirus into TDSCs to reduce the expression of LCAD protein. It can be detected by immunofluorescence and WB that the virus was successfully transfected into the cells and the expression of LCAD was reduced (Figure 5E). Through LCAD and MCAD immunofluorescence staining and WB experiments, we found that inhibiting β‐oxidation could decrease the expression of LCAD and MCAD proteins compared to POSTN knockout (Figure 5F,G). Additionally, ALP and ARS staining also confirmed that inhibiting β‐oxidation could reverse the inhibitory effect of POSTN knockout on osteogenesis (Figure 5H). Runx2 immunofluorescence staining and Micro‐CT measurements also proved that inhibiting β‐oxidation could mediate osteogenic effects both in vivo and in vitro. (Figure 5I). At the same time, WB and immunofluorescence results showed that after POSTN knockout, inhibiting β‐oxidation could increase the expression of osteogenic‐related proteins (Figure S5A, Supporting Information). Safranine O staining and H&E staining showed that after POSTN knockout, inhibiting β‐oxidation could induce osteogenesis (Figure S5C, Supporting Information).

### 2.6 POSTN Enhances Osteogenic Propensity by Binding to PTK7

**Figure 6:**
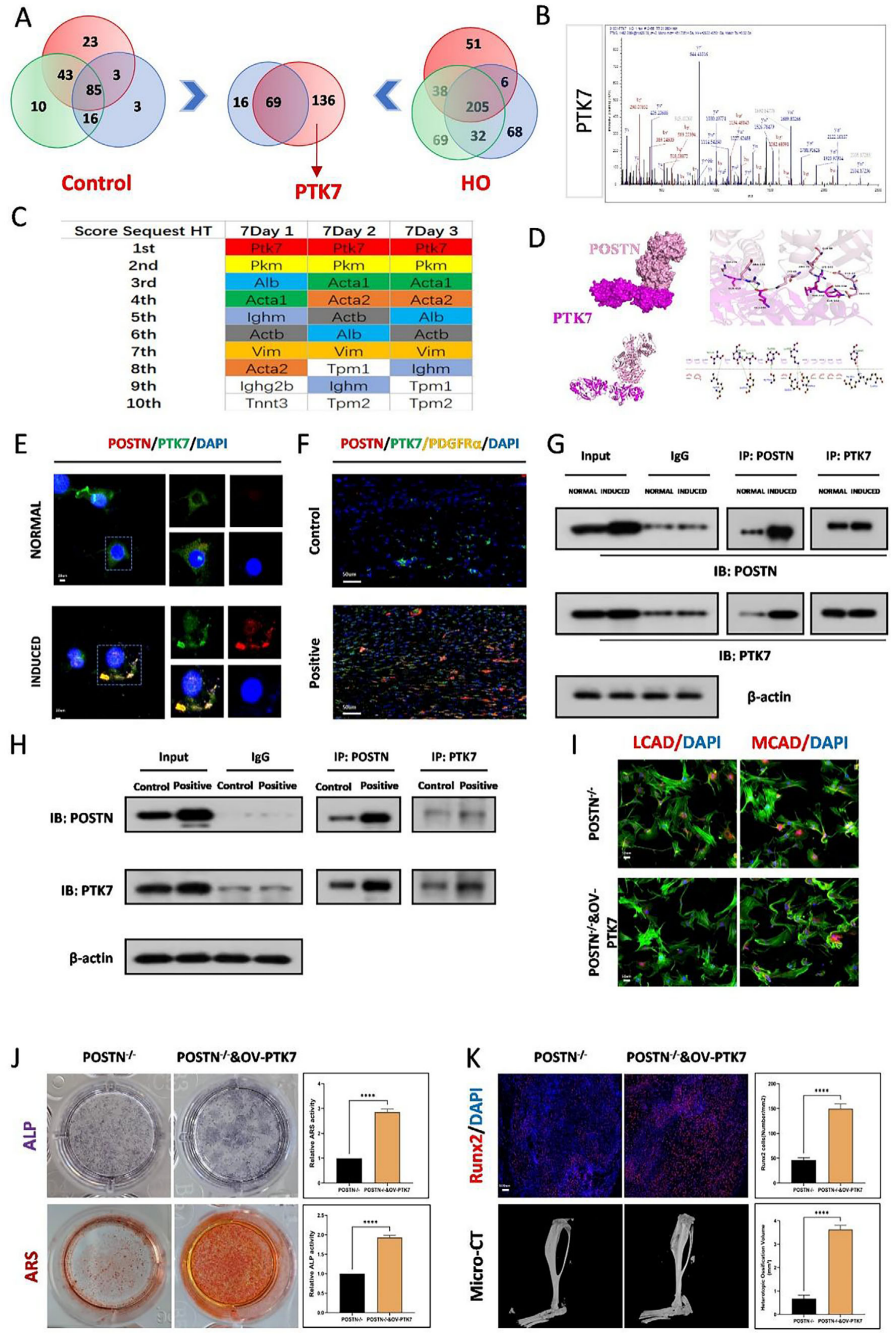
POSTN enhances osteogenic propensity by binding to PTK7. A) Mass spectrometry analysis was used and presented through a Venn diagram to detect the potential molecules that showed increased binding to POSTN in the disease model between the sham group and the tendon injury group at 7 days, *N* = 3. B) Mass spectrum of the binding between PTK7 and POSTN. C) According to the Score Sequest HT, PTK7 ranks first among the molecules that bind to POSTN in the heterotopic ossification model. D) Docking images showing the predicted binding position of POSTN and PTK7 protein. E) IF staining showed co‐localization of POSTN and PTK7 in the cytoplasm of TDSCs, *N* = 3. F) IF staining showed co‐localization of POSTN and PTK7 in the heterotopic ossified tissue, *N* = 6. G) Co‐IP analysis was used to verified the bind relationship between POSTN and PTK7 in the osteogenic induced TDSCs, *N *= 3. H) Co‐IP analysis was used to verified the bind relationship between POSTN and PTK7 in the heterotopic ossified tissue, *N* = 3. I) Immunofluorescence staining indicated that overexpression of PTK7 could upregulate the protein expression levels of LCAD and MCAD in the absence of POSTN in vitro, *N* = 3. J) ALP and ARS staining was used to detect the osteogenesis of TDSCs in the POSTN‐/‐ and POSTN‐/‐&OV‐PTK7 groups, *N* = 6, ****p < 0.0001. K) Immunofluorescence staining for the Runx2 of tendons and micro‐CT analysis of HO formation in the POSTN‐/‐ and POSTN‐/‐&OV‐PTK7 groups, *N* = 6, ****p < 0.0001.

Subsequently, to identify the downstream interacting proteins of POSTN, we conducted mass spectrometry analysis. Through the score sequest HT scoring, it was found that PTK7 protein showed a very significant binding to POSTN in the disease model of HO (**Figure** **6**A–C). Similarly, the use of the molecular docking software for prediction also confirmed the direct physical binding ability between POSTN and PTK7(Figure 6D). Both cellular and tissue immunofluorescence staining showed co‐localization of POSTN and PTK7 in the cytoplasm (Figure 6E,F). Furthermore, we conducted co‐immunoprecipitation analysis and ensure a direct interaction between POSTN and PTK7 both in vitro (Figure 6G) and in vivo (Figure 6H). Additionally, immunofluorescence staining and WB results indicated that overexpression of PTK7 could downregulate the protein expression levels of LCAD and MCAD in the absence of POSTN (Figure 6I; Figure S6A,B, Supporting Information). ALP and ARS staining also confirmed that overexpression of the PTK7 protein could reverse the inhibitory effect of POSTN knockout on osteogenesis (Figure 6J), as well as WB experiments and immunofluorescence staining (Figure S6C,D, Supporting Information). Concurrently, Runx2 immunofluorescence staining and micro‐CT measurements similarly demonstrated that overexpression of the PTK7 protein could reverse the suppression of osteogenic effects caused by POSTN knockout (Figure 6K), as well as Safranine O staining and H&E staining (Figure S6E, Supporting Information).

### 2.7 PTK7 Mediates the Osteogenic Effect in TDSCs through AKT

**Figure 7:**
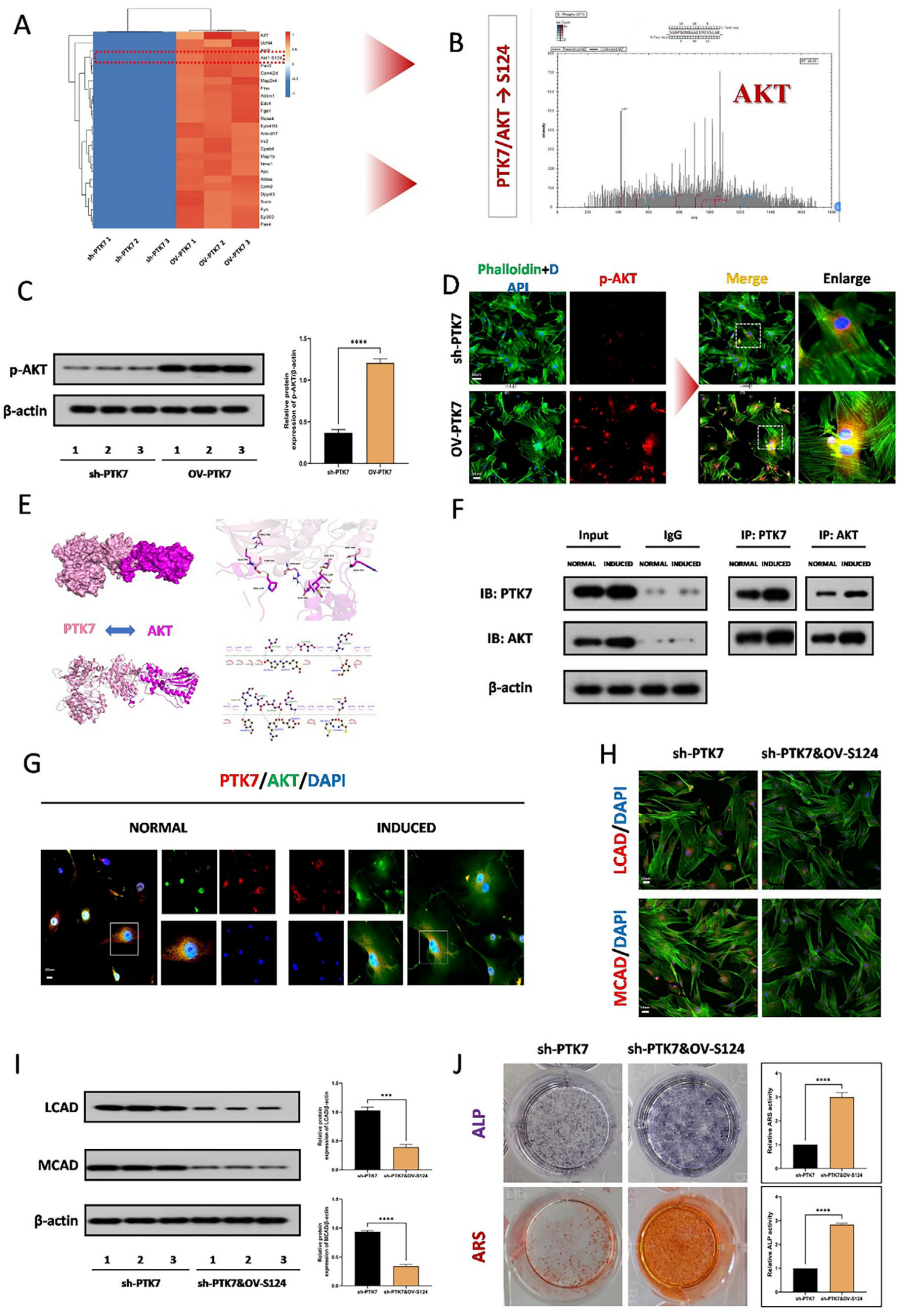
PTK7 mediates the osteogenic effect in TDSCs through AKT1. A) Phosphorylation sequencing analysis was used to detect the effects of PTK7 on downstream proteins., *N* = 3. B) The mass spectrometry diagram showing the increased phosphorylation at the S124 site of the AKT protein C) WB analysis was used to detect the expression of PTK7 could regulate the protein expression levels of p‐AKT in the TDSCs, *N* = 3, **** *p* < 0.0001. D) Immunofluorescence staining indicated that overexpression of PTK7 could upregulate the p‐AKT in the TDSCs, *N* = 3. E) Docking images showing the predicted binding position of PTK7 and AKT protein. F) Co‐IP analysis was used to verified the bind relationship between PTK7 and AKT in the osteogenic induced TDSCs, *N* = 3. G) Immunofluorescence staining indicated that overexpression of PTK7 could upregulate the p‐AKT in the TDSCs, *N* = 3. H) Immunofluorescence staining for LCAD and MCAD of TDSCs in the sh‐PTK7 and sh‐PTK7&OV‐S124 groups, *N* = 3. I) WB analysis was used to detect the expression of LCAD and MCAD proteins of TDSCs in the sh‐PTK7 and sh‐PTK7&OV‐S124 groups, *N* = 3, ***p < 0.001, ****p < 0.0001. J) ALP and ARS staining were used to detect the osteogenesis of TDSCs in the sh‐PTK7 and sh‐PTK7&OV‐S124 groups, *N* = 6, ****p < 0.0001.

Furthermore, by comparing the phosphorylation sequencing results after overexpressing and reducing the PTK7 protein, it was found that there was a significant increase in the phosphorylation of the serine residue at position 124 of AKT in PTK7‐overexpressing TDSCs. (**Figure** **7**A,B). Since the S124 site of AKT is a phosphorylation site, we further examined the phosphorylation level of AKT. WB and immunofluorescence results showed that overexpression of PTK7 significantly increased the phosphorylation level at the S124 site of AKT (Figure 7C,D). Furthermore, we delved into the specific mode of interaction between PTK7 and AKT. Similarly, the use of molecular docking has confirmed the direct physical binding ability between PTK7 and AKT (Figure 7E). Co‐immunoprecipitation experiments also confirmed the interaction between PTK7 and AKT in TDSCs (Figure 7F). Similarly, immunofluorescence staining also showed co‐localization of PTK7 and AKT (Figure 7G). The above results indicate that PTK7 directly binds to AKT and increases the phosphorylation level at the S124 site of AKT. Subsequently, in vitro, after transfection of the lentiviruses targeting AKT (S124), a reduced β‐oxidation was confirmed by seahorse OCR (Figure S7A, Supporting Information). The corresponding results were also shown by immunofluorescence staining for LCAD and MCAD both in vitro (Figure 7H) and in vivo (Figure S7B, Supporting Information) as well as Western blot analysis (Figure 7I). In vitro, ALP and ARS staining confirmed that after knocking down PTK7, overexpression of phosphorylated AKT at the S124 site could promote osteogenic effects (Figure 7J), as well as immunofluorescence staining and WB experiments (Figure S7C,D, Supporting Information). In vivo, Runx2 immunofluorescence staining, Safranine O staining, micro‐CT measurements, and H&E staining also confirmed that overexpression of phosphorylated AKT at the S124 site could reverse the inhibitory effect of knocking down PTK7 on osteogenesis (Figure S7E,F, Supporting Information).

### 2.8 POSTN Promotes the Osteogenic Transition of TDSCs by Mediating the Phosphorylation of AKT at the S124 Site

**Figure 8:**
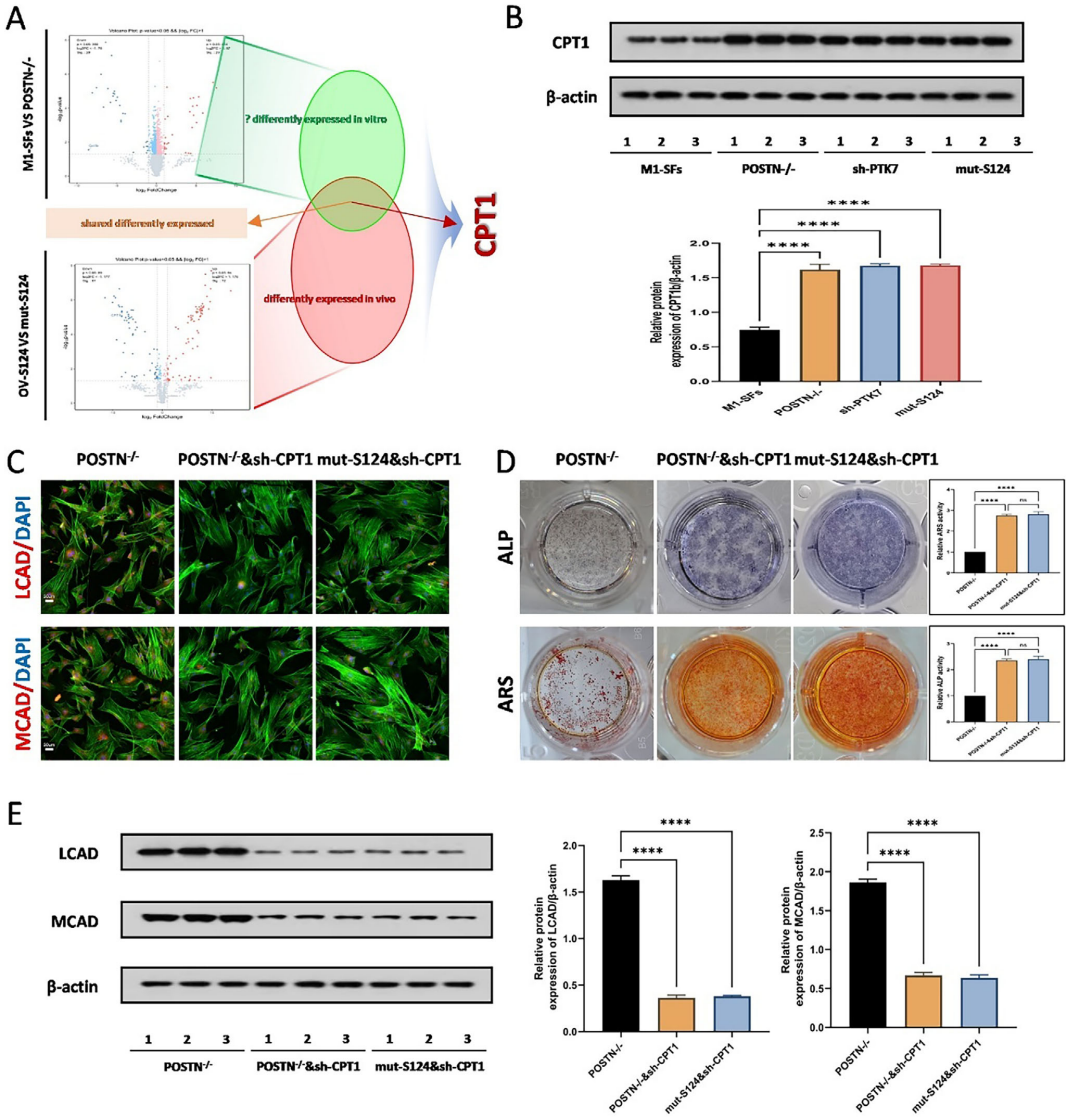
POSTN promotes the osteogenic transition of TDSCs by mediating the phosphorylation of AKT at the S124 site. A) High‐throughput sequencing was performed between TDSCs treated with SFs derived from M1 macrophages and SFs derived from M1 macrophages with the POSTN protein knocked out respectively and between overexpressed phosphorylation at the AKT S124 site and the mutated S124 site respectively. An intersection Venn diagram was drawn. B) WB analysis was used to detect the expression of CPT1 proteins of TDSCs in the M1‐SFs, POSTN‐/‐, sh‐PTK7, and mut‐S124 groups, *N *= 3, ****p < 0.0001. C) Immunofluorescence staining for LCAD and MCAD of TDSCs in the POSTN‐/‐, POSTN‐/‐&sh‐CPT1 and mut‐S124&sh‐CPT1 groups, *N *= 3. D) ALP and ARS staining were used to detect the osteogenesis of TDSCs in the POSTN‐/‐, POSTN‐/‐&sh‐CPT1 and mut‐S124&sh‐CPT1 groups, *N* = 6, ****p < 0.0001. E) WB analysis was used to detect the expression of LCAD and MCAD proteins of TDSCs in the POSTN‐/‐, POSTN‐/‐&sh‐CPT1 and mut‐S124&sh‐CPT1 groups, *N* = 3, ****p < 0.0001.

Subsequently, we further explored the underlying mechanism by which POSTN affects the phosphorylation of AKT to promote the osteogenic differentiation of TDSCs. Proteomics sequencing was carried out between the TDSCs induced respectively by the SFs from M1‐derived macrophages and the macrophages derived from POSTN‐knockout M1 macrophages. When combining the results of proteomics sequencing between TDSCs with AKT overexpressed at the S124 site and those with the S124 site mutated, bioinformatics analysis identified the commonly differentially expressed proteins in the above sequencing, and the results showed that the protein CPT1 ranked among the top 10. (**Figure** **8**A). Similarly, a significant increase in CPT1 protein levels was further confirmed by WB in the absence of POSTN, after knocking down PTK7, and upon mutant of AKT S124 (Figure 8B). Immunofluorescence staining and WB results showed that knocking down CPT1 after POSTN knockout could reverse the expression levels of LCAD and MCAD, a result consistent with the outcome after overexpressing AKT and knocking down CPT1 (Figure 8C,E). We then performed ALP and ARS staining, and the results showed that knocking down CPT1 after POSTN knockout could increase osteogenic levels, as well as upon the mutant of AKT S124 and knocking down CPT1 could also increase osteogenic levels (Figure 8D). Furthermore, we observed through immunofluorescence and WB that knocking down CPT1 after POSTN knockout could reverse the inhibition of osteogenic‐related proteins after POSTN knockout, a result that also applies to overexpressing phosphorylation site‐mutated AKT (Figure S8A,B, Supporting Information). In vivo, H&E staining, Safranine O staining, Runx2 immunofluorescence staining, and Micro‐CT measurements supported that decreasing CPT1 after POSTN knockout led to the formation of heterotopic bone, and overexpressing phosphorylation site‐mutated AKT and knocking down CPT1 had the same phenomenon (Figure S8C,D, Supporting Information). Further, based on previous literature reports, we analyzed the AMPK/ACC/CPT1 pathway through WB detection.^[^ ^23^ ^]^ The results indicate that phosphorylation at the AKT124 site activates the AKT activation site at 473, which promotes phosphorylation at the AMPK S485 site, thereby inhibiting the activation of AMPK. This, in turn, triggers a cascade effect, reducing phosphorylation at the ACC S79 site, leading to an increase in the synthesis of malonyl – CoA in vivo and thereby inhibiting CPT1(Figure S9, Supporting Information)

## 3 Discussion

HO is characterized by endochondral bone formation within the tendon and is a major histological feature in the late stages of tendon injuries.^[^ ^24^ ^]^ Calcification of the achilles tendon can occur unilaterally or bilaterally, initiating at the tendon body or the enthesis, leading to the formation of a solid, painful mass. Therefore, inhibiting the ossification process can improve the quality of life for patients. The osteogenic differentiation of TDSCs is largely regulated by the microenvironment in which they reside, especially under pathological conditions such as injury, burns, and amputation surgery. In these cases, a large number of perivascular immune cells appear in the surrounding areas of the heterotopic ossification lesion, such as skeletal muscle and connective tissue, followed by immune cell infiltration and destruction of connective tissue structure, creating conditions for HO formation.^[^ ^25^ ^]^ Macrophages are crucial for tissue repair as they clear cellular debris, secrete growth factors to stimulate cell proliferation and angiogenesis, and orchestrate the interaction among various cells. They interact with other cells involved in repair, like stem cells, guiding their differentiation and integration into the regenerating tissue. Therefore, several studies have developed therapeutic materials targeting macrophages in osteogenic environments to regulate the balance of osteogenic differentiation.^[^ ^26^, ^27^, ^28^ ^]^ In this study, transcriptional profiling and GSEA analysis reveal a significant upregulation of M1 macrophage polarization in the injured tissue model compared to the normal state. In this study, the polarization level of M1 was reduced by knocking out the NF‐κB  gene, consistent with the results of others.^[^ ^29^ ^]^ Correspondingly, the volume of HO was also significantly decreased. Although, macrophages play a crucial role in tissue remodeling and initiating HO formation, yet the precise downstream mechanisms remain to be further explored. According to what previous scholars have discovered, many experimental studies have shown that paracrine plays a crucial role in the communication between immune cells and stem cells.^[^ ^30^ ^]^ M1 macrophages polarize under the influence of pro‐inflammatory factors such as LPS and IFN‐γ, highly expressing IL‐12 and IL‐23, producing a large amount of pro‐inflammatory factors such as TNF‐α and IL‐6 which promote inflammation and pathogen clearance.^[^ ^14^ ^]^ Moreover, it has also been reported that M1 polarized macrophages can transfer specific miRNAs and proteins to stem cells, which may dysregulate the intracellular signaling cascades of stem cells and cause them to lose normal differentiation cues, thus leading to abnormal differentiation phenotypes.^[^ ^31^ ^]^ Then which components secreted by M1 polarized macrophages regulate the differentiation of TDSCs? In this study, by stimulating with two components both in vivo and in vitro respectively, we found that SFs are the more dominant regulatory components.

Paracrine refers to the signal molecules secreted by cells, which diffuse through the intercellular space and act on the neighboring target cells to regulate the physiological functions of these cells.^[^ ^30^ ^]^ Several studies have found that macrophages communicate with other cells through paracrine, achieving the regulation of osteogenic differentiation.^[^ ^19^, ^32^ ^]^ We discovered for the first time that M1 macrophages mediate traumatic HO formation by secreting POSTN. Lin and colleagues found that TDSCs with Mohawk (MKX) knockout exhibited overactivation of POSTN during osteochondral formation.^[^ ^33^ ^]^ Our study complements this finding, showing that POSTN in TDSCs is not only regulated by MKX but also by SFs containing POSTN secreted by M1 macrophages. In this study, we found that POSTN was significantly overexpressed in the mouse model of HO and was significantly enriched in the SFs of M1 polarized macrophages. Furthermore, we discovered that POSTN can enhance FAO, a process mainly enhanced by the upregulation of expression of proteins related to FAO, such as LCAD and MCAD. Additionally, scholars have found in studies of tendon disease complicated by diabetes that high glucose enhances the osteogenic differentiation capacity of TDSCs, inhibits tenogenic differentiation capacity, and promotes abnormal differentiation of TDSCs.^[^ ^34^ ^]^ Therefore, targeting the metabolic reprogramming of TDSCs is also an important therapeutic target, as previous studies have shown that intervening in the FAO process of cardiac stem cells can lead to cardiac regeneration.^[^ ^35^ ^]^

Interestingly, in previous studies, scholars have found that in a tendon injury model established by excising the central one‐third of the Achilles tendon from the distal apex of the muscle to the calcaneal insertion, exogenous supplementation of POSTN protein promotes the differentiation of TDSCs toward the tendon lineage.^[^ ^36^ ^]^ In contrast, in a HO model established by Achilles tendon transection combined with dorsal scalding, POSTN promotes osteogenic differentiation of TDSCs. It is well‐known that heterotopic bone development requires the synergistic action of three key factors: osteogenic progenitor cells, molecular signals triggering heterotopic bone formation, and a suitable microenvironment.^[^ ^37^, ^38^ ^]^ Among them, the suitable microenvironment is crucial for HO. Post‐traumatic inflammation is known to induce HO,^[^ ^16^ ^]^ with various immune cells including macrophages, mast cells, and lymphocytes, as well as inflammatory cytokines such as TNF‐α and IL‐1β, participating in the development of HO.^[^ ^39^, ^40^, ^41^, ^42^, ^43^, ^44^ ^]^ Previous studies have reported that dorsal scalding significantly increases macrophage recruitment and inflammation response in the tendon injury site, thereby enhancing the inflammatory response.^[^ ^15^, ^45^ ^]^ To investigate the divergent effects of POSTN protein on TDSCs differentiation in the two models, we established both models and compared their microenvironments (Figure S10A, Supporting Information). The results of WB showed that in the HO model, local macrophage marker F4/80, M1 macrophage marker CD86, and inflammatory cytokine markers IL‐1β and TNF‐α were all higher in simple Achilles tendon transection than in the tendon injury model, with dorsal scalding further exacerbating these differences (Figure S10B, Supporting Information). Additionally, even after macrophage depletion with clodronate, inflammatory factors IL‐1β and TNF‐α remained significantly higher in the HO model than in the tendon injury model (Figure S10C, Supporting Information). POSTN protein expression was lower in the simple tendon transection model than in the tendon injury model but significantly increased after dorsal scalding. These results indicate that the inflammatory microenvironment in the HO model is much more severe than that in the tendon injury model, and POSTN protein is predominantly derived from migrating macrophages. Previous studies have shown that POSTN promotes osteogenic differentiation of stem cells in inflammatory microenvironments. Further, when we injected the supernatant derived from POSTN‐knockout M1 macrophages into the tendon injury model and supplemented with recombinant POSTN protein, safranin‐O and fast green staining and immunofluorescence staining revealed the presence of HO tissue at the tendon stumps (Figure S10D, Supporting Information). In the previous literature, the authors also found that POSTN protein promotes osteogenic differentiation of TDSCs when cultured in osteogenic medium.^[^ ^36^ ^]^ Together, these findings indicate that the role of POSTN protein in regulating TDSCs differentiation is highly microenvironment‐dependent. Therefore, we hypothesize that although both models involve tendon injury, POSTN interacts with distinct molecular proteins on TDSCs in the inflammatory microenvironment of the HO model, activating different downstream pathways and ultimately leading to divergent differentiation fates of TDSCs.

Our study further clarified the downstream molecules and signaling pathways regulated by POSTN using experimental methods such as mass spectrometry and found that POSTN could directly interact with PTK7. PTK7, also known as colon cancer kinase 4 (CCK4), is a receptor protein tyrosine kinase (RPTK)‐like molecule and a member of the catalytically defective receptor protein tyrosine kinase family, which is upregulated in various tumors.^[^ ^46^ ^]^ PTK7 has been reported to participate in the transduction of various cellular pathways, including cell migration, proliferation, and survival. A classic pathway is that PTK7 may pair with VEGF‐R1 to form a dimer, inducing the phosphorylation of intracellular proteins FAK and AKT, thereby regulating cell migration, proliferation, and survival.^[^ ^47^ ^]^ Previous studies have found that PTK7 can enhance the phosphorylation level of AKT protein. However, scholars have not explored the specific mechanism and sites of its phosphorylation. In our study, we also found that the protein complex formed by POSTN and PTK7 can increase the level of AKT phosphorylation at the S124 site. Several studies have pointed out that the upregulation of the PI3K/AKT signaling pathway is involved in the exacerbation of tendon HO. Chen's study pointed out that inhibitors targeting the PI3K/AKT pathway will be a promising method for treating hematogenous tendon HO.^[^ ^48^ ^]^ Andreas and his team found that by inhibiting the activity of protein kinase AKT and ribosomal S6 kinase, the synthesis of type I collagen in tendon and ligament tissues under mechanical load can be reduced.^[^ ^49^ ^]^ It is generally believed that the AKT signal promotes lipid synthesis, inhibits fat decomposition, and β‐oxidation. However, the precise involvement of AKT in balancing FAOs and glucose oxidation shows its more complex role.^[^ ^50^ ^]^ It is described in the article that AKT is a key molecule in the insulin signaling pathway and can phosphorylate multiple substrates to regulate cell metabolism. As a key enzyme in fatty acid oxidation, the expression of CPT1 may be indirectly affected by AKT protein via the AMPK/ACC/CPT1 pathway.^[^ ^51^ ^]^ In this study, we carried out further detailed verification on this pathway. Specifically, AKT can phosphorylate AMPK at residue S485, reducing the activity of AMPK and decreasing its phosphorylation of the ACC protein at residue S79. This leads to an increase in the synthesis of malonyl‐CoA in the body, which in turn inhibits carnitine acyltransferase‐1 (CPT‐1) and ultimately inhibits the oxidation of long‐chain acyl‐CoA as it enters the mitochondria from the cytoplasm. In addition, apart from its effect on AKT, the role of the POSTN protein may be far more than that. There have also been reports in previous literature about its inhibitory effect on the key protein of fatty acid oxidation, the PPARγ protein.^[^ ^52^ ^]^ Therefore, more research is needed in the future to clarify the role and mechanism of POSTN in the regulation of metabolism during the HO process.

## 4 Conclusion

Our study reveals that macrophage polarization toward the M1 phenotype after tendon injury inhibits the enhancement of fatty acid oxidation metabolism in TDSCs, leading to HO formation. Mechanistically, during the progression of HO, the extracellular vesicles secreted by macrophages and containing periostin (POSTN) synergistically bind to protein tyrosine kinase (PTK), which results in an increase in the phosphorylation level of AKT in TDSCs. Our findings provide new insights into the microenvironment and cellular dialogue during HO formation and point to new directions for HO treatment.

## 5 Experimental Section

### Reagents and Materials

Fetal bovine serum (FBS) and alpha‐minimum essential medium (α‐MEM) were purchased from Gibco (Carlsbad, CA). Penicillin/streptomycin was purchased from HyClone (Logan, UT, USA). M‐CSF (Cat# C756) was purchased from Novoprotein (Suzhou, China). LPS (Cat# L2880) was purchased from Sigma‐Aldrich (St.Louis, MO, USA). Adeno‐associated virus (AAV) and adeno virus (AV) for downregulating long‐chain acyl‐CoA dehydrogenase (sh‐LCAD), Protein Tyrosine Kinase 7(PTK7), and Carnitine Palmitoyl Transferase 1(CPT1), upregulating PTK7, along with their corresponding controls, were purchased from Genomeditech Co., Ltd (Shanghai, China). The CD86 (Cat# 13395‐1‐AP), CD80 (Cat# 66406‐1‐lg), TLR2 (Cat# 66645‐1‐lg), Runx2 (Cat# 20700‐1‐AP), OPN (Cat# 22952‐1‐AP), OCN (Cat# 23418‐1‐AP), CD9 (Cat# 60232‐ 1‐lg), CD81 (Cat# 27855‐1‐AP), ALIX (Cat# 12422‐1‐AP), TSG101 (Cat# 28283‐1‐AP), Calnexin (Cat# 10427‐2‐AP), PTK7 (Cat# 17799‐1‐ AP), LCAD (Cat# 17526‐1‐AP), IL1β (Cat# 226048‐1‐AP), TNFα (Cat# 26162‐1‐AP), AMPK(Cat# 10929‐2‐AP), AKT(, Cat# 80457‐1‐RR) antibodies and p‐AKT(Ser473) (Cat# 66444‐1‐ Ig) were purchased from Proteintech (Chicago, IL, USA). The POSTN (Cat# ab315104), MKX (Cat# ab036400), and SCX (Cat# ab307722) antibodies were purchased from Abcam (USA). The PDGFRα (Cat# AF1062) antibody was purchased from R&D Systems (Minneapolis, MN, USA). The MCAD (Cat# A4567) antibody was purchased from Abclonal (Wuhan, China). The anti‐AMPK‐pS485 (Cat# 2537), anti‐AMPK (Cat# 2532), anti‐ACC (Cat# 3676), and anti‐ACC‐pS79 (Cat# 11 818) were purchased from Cell Signaling Technology (Danvers, MA, USA). The F4/80 (Cat# GB113373) and β‐actin (Cat# GB15001) antibodies were purchased from Servicebio (Wuhan, China). p‐AKT was customized from Genscript (Nanjing, China). The recombinant POSTN protein and its inhibitor HY‐RS16974 were purchased from MedChemExpress (Shanghai, China).

### Animal Models

Wild‐type C57BL/6 (WT) aged six weeks were housed in the Animal Experiment Center of Shanghai Sixth People's Hospital. NFkB global knockout (NFkB−/−) mice and Periostin (POSTN) global knockout (POSTN−/−) mice were obtained from GemPharmatech company (Nanjing, China, NFkB‐/‐| Strain NO. T007133, POSTN‐/‐| Strain NO. T011526). Mice were housed in rooms with free access to food and water, under controlled temperature (21–26 °C) and humidity (50–60%) conditions, with a 12‐hour light‐dark cycle. The primers used for genotyping of NFkB−/− and POSTN−/− mice are listed in Table S1 (Supporting Information). Male mice were anesthetized with 0.25% tribromoethanol at a dose of 125–240 mg kg^−1^. Once the mice were anesthetized, the inner side of the hind limbs was shaved and disinfected, and a 5 mm longitudinal skin incision was made above the posterior lateral aspect of the ankle joint. The Sham group was closed with sutures immediately after the skin was incised; the HO group had the Achilles tendon bluntly dissected with ophthalmic forceps, fully exposed, and transected at the midpoint. The tendon ends were clamped with a hemostat without suturing the tendon, and the skin was closed. Subsequently, the dorsal hair of the mice was shaved over an area of 2 cm × 3 cm, and a 2 × 2 × 3 cubic centimeter aluminum block weighing 35 g at 60 °C was placed on the exposed dorsal skin of the mice for 17 s to cause a 30% total body surface area burn, the Sham group did not undergo this procedure. The wounds were disinfected with povidone‐iodine, and the mice were placed on a heating pad and returned to their cages after they regained consciousness. Mice given M1 macrophage exosomes were injected locally into the tendon after surgery, while control mice were injected with PBS. To extract the HO tissue formed 7 days after injury from a tendon transection model: first, the animal was anesthetized and euthanized. Used an electric shaver to carefully remove hair from the surgical area (the Achilles tendon region of the mouse hindlimb). Disinfected the depilated area with 75% alcohol three times, with each disinfection range gradually expanding from the inside out. After disinfection, a sterile surgical drape was spread. A 1–2 cm incision was made along the long axis of the tendon at the injured site using an 11‐blade scalpel, with the blade perpendicular to the skin; performed the action gently to avoid excessive damage to subcutaneous tissue. Used ophthalmic curved forceps to bluntly dissect the subcutaneous tissue, separated the skin from the underlying fascia and muscle, and fully exposed the injured tendon area. At this point, the broken end of the injured tendon could be seen. Used blunt‐tipped forceps to bluntly dissect and removed the adherent normal tissue, then used microscissors to obtain the HO tissue. The animal experiments complied with the regulations on the administration of experimental animals in China and were approved by the Ministry of Science and Technology. Researchers were blinded to group allocation during data analysis. A tendon injury model analogous to the HO model was established based on previous literature to compare the distinct roles of POSTN protein in the two models.^[^ ^36^ ^]^ The central one‐third of the Achilles tendon (≈4 mm in width) was removed from the distal apex of the muscle to the insertion of the calcaneus.

### High‐Throughput Sequencing

The soft tissue at the Achilles tendon underwent whole transcriptome genome sequencing (provided by Shanghai OE Biotech. Co., Ltd). RNA was extracted using Trizol (USA, Invitrogen Corporation), and after quality inspection with Agilent 2200, polyA RNA was obtained using the Dynabeads mRNA Purification Kit (Thermo, Cat.1264684). The obtained poly(A) RNA was fragmented using RNAase III and a sequencing library was constructed using the Ion Total RNA‐seq Kit v2. The constructed library, after passing the quality inspection with Agilent 2200, was sequenced using the Ion OneTouch. The raw data obtained from sequencing were first subjected to adapter sequence removal, followed by the removal of sequences containing more than 5% ambiguous bases, and finally the removal of sequences with more than 20% of base qualities less than 13. The filtered data were aligned to the mm9 genome using MapSlice. The resulting expression matrix was subjected to differential gene expression analysis using the R package DESeq2. Similarly, the proteomic data analysis was accomplished by Shanghai OE Biotech Co., Ltd., located in Shanghai, China. The entire analytical process was executed with a timsTOF Pro mass spectrometer (manufactured jointly by Bruker and Thermo) that was furnished with an Easyspray source (from Thermo, USA). Samples were introduced into an EASY‐nLCTM 1200 system (of Thermo, USA) via a C18 column (having a dimension of 15 cm × 75 µm). The flow rate was maintained at 300 nL min^−1^, and the linear gradient was configured in the following manner: during the period from 0 to 20 min, the proportion of phase B was adjusted from 5% to 22%; from 20 to 24 min, it ranged from 22% to 37% of phase B; from 24 to 27 min, it changed from 37% to 80% of phase B; and from 27 to 30 min, it remained at 80% of phase B. The ion mobility was set within the range of 0.7 to 1.3 Vs cm^−^^2^, and the collision energy spanned from 20 to 59 eV. The MS/MS spectra were recorded between 100 and 1700 m/z. The Spectronaut Pulsar 18.4 software (developed by Biognosys, Swiss) was utilized to search the MS/MS spectra against the UniProt database of Mus Musculus. The specific parameters for the database search were established as follows: for fixed modifications, Carbamidomethyl (C) was set; for variable modifications, Oxidation (M) and Acetyl (Protein N‐term) were specified; the digestion enzyme was Trypsin; the Precursor *Q*‐value cutoff was 0.01; the Protein *Q*‐value cutoff was 0.01; the number of allowed missed cleavages was 2; and the quantification MS level was MS2Heatmaps of gene expression were plotted using the R package heatmap. Gene Set Enrichment Analysis (GSEA) was performed using the R package clusterprofiler.

### Western Blot

Cells or tissues were lysed using RIPA lysis buffer containing protease and phosphatase inhibitors (USA, Thermo Company), lysed on ice for 10 min, centrifuged at 4 °C and 14 500 rpm for 20 min, and the supernatant was collected. Protein concentration was determined using the BCA method according to the instructions of the BCA kit (Servicebio, Wuhan, China). Based on the measured protein concentration, 5× loading buffer was added, and proteins were boiled and denatured for 10 min. Each well was loaded with 15–25 µg of protein and subjected to constant voltage 100 V electrophoresis. Subsequently, proteins were transferred to a PVDF membrane (Servicebio, Wuhan, China) at a constant current of 200 mA. The PVDF membrane was washed three times with TBST. After blocking the PVDF membrane with 5% skim milk at room temperature for 2 h, it was incubated with the primary antibody overnight at 4 °C on a shaker. The following day, the PVDF membrane was washed three times with TBST, and then incubated with the corresponding secondary antibody (USA, Proteintech Company) at room temperature on a shaker for 2 h. The PVDF membrane was washed three times with TBST. Bands were developed using the ECL chemiluminescence method (USA, Proteintech Company), and the grayscale of the bands was quantified using Image J software.

### Histological, Immunohistochemical, and Immunofluorescence Staining

Achilles tendon heterotopic ossification tissues were harvested and fixed in 10% formalin for 24 h, then decalcified with 10% Ethylenediaminetetraacetic acid (EDTA) for 2 weeks. Finally, the tissues were dehydrated through a graded ethanol series, cleared in xylene, and embedded in paraffin to produce 4 µm sections. Tissue paraffin sections were baked at 72 °C for 30 min, and deparaffinized in xylene I, xylene II, xylene III, anhydrous ethanol I, anhydrous ethanol II, 95% ethanol, 90% ethanol, and 80% ethanol for 10 min each, followed by gentle rinsing with water.

For Hematoxylin and Eosin (H&E) staining, the sections were immersed in hematoxylin stain for 10 min, rinsed with water for 2 min. Then, they were immersed in 1% hydrochloric acid alcohol for 1 s, and rinsed three times with water. Afterward, the sections were immersed in eosin stain for 20 s. They were rinsed once with tap water. The sections were soaked in 80% ethanol for 20 s, checked for decolorization, and after completion of decolorization, soaked in 90% ethanol for 15 s, 100% ethanol for 15 s, a mixture of ethanol and xylene (1:1) for 1 min, and xylene for 2 min. The sections were then mounted with neutral resin and air‐dried in a fume hood. Finally, the staining effect was observed using an upright optical microscope. Safranin O staining solution was applied for 5 min, followed by a brief rinse with tap water. The sections were then soaked in 95% ethanol for 3 s, anhydrous ethanol I for 3 s, anhydrous ethanol II for 1 min, xylene for 5 min, and mounted with neutral resin. The staining effect was observed under a microscope. For immunofluorescence staining (IF), after deparaffinization, the sections were washed with PBS for 5 min, and boundaries were drawn with a histology pen, with antibody names marked in blank areas. Sections were treated with 0.4% pepsin at 37 °C for 30 min for antigen retrieval. Then, they were washed three times with PBS, treated with 0.1% Triton‐PBS, placed in a humid chamber at room temperature for 15 min, and washed three times with PBS. A 5% BSA blocking solution was applied to the sample surface and incubated at 37 °C for 60 min. After washing off the blocking solution, the primary antibody was applied to the sample surface and incubated overnight at 4 °C. The following day, the sections were washed with PBS‐Tween, and the secondary antibody was added and incubated at room temperature for 2 h. After washing with PBS, the sections were mounted with an immunofluorescence mounting medium containing 4′,6‐diamidino‐2‐phenylindole (DAPI). The stained slides were scanned using a Panoramic digital scanner (Pannoramic P250; 3DHISTECH).

### Primary Culture of Bone Marrow‐Derived Macrophages (BMDM)

Male WT mice aged 6 weeks were sacrificed, and the intact femurs and tibias were dissected free of soft tissue. The bones were held with tweezers, and the femurs were cut at the junctions of the greater and lesser trochanters and the medial epicondyle, while the tibias were cut below the tibial plateau and above the ankle joint to obtain the tubular bones. The bone marrow cavity was flushed with 4 °C PBS, and the marrow was collected and blown to prepare a bone marrow cell suspension. The bone marrow cell suspension was filtered through a 200 µm mesh and centrifuged at 1000 rpm for 5 min, after which the supernatant was discarded. The cells were resuspended in red blood cell lysis buffer at 4 °C and lysed to remove red blood cells. After centrifugation at 1000 rpm for 5 min, the supernatant was discarded, and the cells were washed twice with PBS. The cell suspension was then plated in culture dishes. Pre‐warmed culture medium supplemented with 20 ng mL^−1^ of M‐CSF was added to induce differentiation. The cultures were incubated at 37 °C with 5% CO2 for 7 days to induce differentiation into M0 macrophages. After induction into M0, 100 ng mL^−1^ of LPS was added to induce polarization toward the M1 phenotype.

### Flow Cytometry Staining of Macrophage

Prepared BMDM (M0) cells (5 × 10^5 ^cells) were resuspended in 100 µL of staining buffer in a test tube. One microgram of Anti‐PacBlue‐F4/80 and 1 µg of Anti‐PE‐CD86 were added to the M0 suspension for surface antibody staining, and the cells were incubated on ice in the dark at 4 °C for 20 min. The cells were then washed with 2 mL of cell staining buffer, centrifuged at 350 g for 5 min, and this washing step was repeated three times. The pelleted cells were resuspended in 500 µL of fixing solution and incubated on ice in the dark at 4 °C for 20 min, followed by centrifugation, and the supernatant was discarded. The pelleted cells were then washed with 2 mL of permeabilization buffer, centrifuged for 5 min, the supernatant was discarded, and this washing step was repeated three times with centrifugation. Finally, the cells were resuspended in 500 µL of staining buffer and analyzed on a flow cytometer. Data analysis was performed using Flow Jo v10.0.7 software.

### Micro‐CT Scanning

The Micro‐CT imaging system produced by Aloka Company in Japan (model: LCT‐100S) was used to perform tomographic scanning imaging on the tissue below the knee joint. The scanning parameters included standard imaging mode, with air CT value set at −1000, bone CT value set at 1000, scanning voltage at 60 kV, power at 30 W, current at 150 µA, and slice thickness at 10 microns. Subsequently, Mimics 21.0 software was utilized to perform 3D reconstruction and imaging recording of the bone quality around the lower limb joints and Achilles tendon. The focus of the analysis was on the bone spurs in the Achilles tendon area. To measure the bone volume, CTan software (Bruker Version 1.15.4.0+) was employed. Any dense masses within the soft tissue with Hounsfield units exceeding were regarded as the indicative markers of heterotopic ossification (HO) according to this software.

### Isolation and Culture of Tendon‐Derived Stem Cells (TDSCs)

Male WT mice aged 6 weeks were sacrificed, and the hind limb skin was disinfected. The skin of the hind limbs was cut and bluntly separated to expose the Achilles tendon. The Achilles tendon and its upper and lower attachment areas were excised. The tendon was uniformly cut into ≈1 mm pieces and then centrifuged at room temperature at 1500 rpm for 3 min. The supernatant was discarded, and the tendon pieces were digested with a final concentration of 3 mg mL^−1^ Type I Collagenase (USA, Sigma Company) and 4 mg mL^−1^ Dispase enzyme (USA, Sigma Company) at 37 °C for 30 min. An appropriate amount of culture medium was added to stop the digestion, mixed well, and then centrifuged at room temperature at 1500 rpm for 5 min. After discarding the supernatant and resuspending the cell pellet, the cell suspension was plated in culture dishes for cultivation. The cells were allowed to grow until they reached 80–90% confluence, after which the culture medium was replaced with osteogenic differentiation medium (USA, Gibco Company) for osteogenic induction.

### Co‐Culture Assay

A Transwell chamber was selected with a 0.4 µm pore size (USA, Corning Company), and then seeded BMDMs in the upper chamber. In the lower chamber, added TDSC cells and osteogenic culture medium, with a seeding ratio of BMDMs to TDSC cells of 4:1.

### Osteogenic Differentiation and Alkaline Phosphatase (ALP) and Alizarin Red S (ARS) Staining of TDSCs

TDSCs were seeded into a 24‐well plate and maintained until they reached 80–90% confluence. The culture medium was then replaced with osteogenic differentiation medium (Cyagen, China) for osteogenic induction. After 7 days of osteogenic induction, the culture medium was discarded, and the cells were rinsed with PBS. The cells were fixed with a mixture of acetone, 40% formaldehyde, and citric acid for 20 s. After discarding the fixative, the cells were washed with deionized water and stained according to the instructions of the ALP staining kit, incubating in the dark at room temperature for 30 min. After rinsing off the staining solution, the cells were observed and photographed under a microscope. The absorbance was measured at 405 nm for quantification. After 21 days of osteogenic induction, the culture medium was discarded, the cells were rinsed with PBS, and stained with a 2% solution of pH 4.2 Alizarin Red S (ARS). The cells were incubated in the dark at room temperature for 45 min. The cells were observed and photographed under a microscope, with undifferentiated osteoblasts (without extracellular calcium deposition) showing a slight red color, while mineralized osteoblasts (with extracellular calcium deposits) exhibited a bright orange‐red color. The deposits were dissolved with a solution containing 0.5* N* HCL and 5% SDS for quantitative analysis, and the absorbance was read at OD405.

### Isolation and Characterization of Extracellular Vesicles (EVs)

Collected the cell culture supernatant from LPS‐induced M1 polarized BMDMs and centrifuge at 4 °C, 300 g for 10 min to remove cell debris and whole cells; discarded the pellet. Centrifuged at 2000 g for 20 min to remove dead cells and debris, then centrifuged at 10 000 g for 30 min; discarded the pellet. Filtered the supernatant through a 0.22 µm filter to remove larger extracellular vesicles, then centrifuged at 120 000 g for 70 min, repeated this step twice. Resuspended the collected EVs pellet in PBS and store at −80 °C. Used a ZetaView nanoparticle tracking analyzer to measure the diameter of the collected particles (exosomal diameter is 50–150 nm). Resuspended the exosomes in 2% soluble polytetrafluoroethylene (PFA), applied a sample to a copper grid, and then added PBS to the edge of the grid. Placed the grid in 1% glutaraldehyde for 5 min, then rinsed in ddH2O. Placed the grid in uranyl oxalate for 5 min, then, working on ice, immersed the grid in a solution of methylcellulose for 10 min. Absorbed excess liquid from the grid with filter paper and air‐dried for 10 min. Finally, examined the grid under a transmission electron microscope at 80 kV to capture images. Used WB analysis to examine the protein expression of CD9, CD81, ALIX, TSG101, F4/80, and calnexin to verify the successful isolation of EVs. After removing the exosomes, the remaining proteins were concentrated in the supernatant.

### Seahorse

The oxygen consumption rate (OCR) of cells was measured using the XF96 Analyzer (Seahorse Bioscience). TDSCs were seeded at a density of 4000 cells per well in an XF 96‐well microplate for osteogenic induction. The night before the experiment, 200 µL of ddH2O was added to each well of the Seahorse XFe96 FluxPak and incubated overnight in a 37 °C incubator without CO_2_. The machine and software were turned on in advance. The night before the experiment, the Seahorse XFe96 Analyzer and Wave software were activated. On the day of the experiment, the ddH2O in the Seahorse XFe96 FluxPak was replaced with XF Calibrant, 200 µL per well. TDSCs were incubated for 1 h in XF assay medium containing 25 mm glucose, 1 mm pyruvate, and 2 mm glutamine at 37 °C in an incubator without CO_2_, and then oxygen consumption analysis was performed. The mitochondrial stress test was measured by sequential injection of isoproterenol (1 mm), ATP synthase inhibitor oligomycin (4 mm), mitochondrial uncoupler carbonyl cyanide 4‐(trifluoromethoxy) phenylhydrazone (FCCP 2 mm), and electron transport inhibitors rotenone and antimycin A (1 mm). After the test, the data were exported, and the cells were quantified using the BCA method. Based on the protein quantification results, the cellular respiration values were normalized and calibrated, and the final results served as the reference values for cellular respiratory capacity.

### Cell Transfection

All virus was purchased from Genomeditech Co., Ltd (Shanghai, China). TDSCs were seeded into a six‐well plate and waited until the cell density reached 70%, then proceeded with lipofection. The adenovirus was collected and purified. Added polybrene at a concentration of 6 µg mL^−1^ to the culture medium and infected TDSCs to overexpress. Replaced the medium after 12 h, and proceeded with subsequent experiments after 48 h. For in vivo studies, knockdown and overexpression of PTK4, LCAD, and AKT S124 were performed with adenovirus transfection, corresponding control AVs were used as control. The indicated AVs were designed and provided from Genomeditech Co., Ltd (Shanghai, China). C57BL/6 mice were injected with 25 µL phosphate buffer saline (PBS) diluted adenovirus (3.5 × 10^10^ viral genomes per injection) in the injury site, once every two weeks for 12 weeks.

### Immunoprecipitation (Co‐IP)

Lysis/wash buffer was added at a ratio of 30 µL per 1 × 10^5^ cells, along with protease inhibitors, mixed well, and incubated on ice for 20 min. Centrifuged at 4 °C, 16 000 × g, for 10 min to collect the supernatant. Then added the antibody to the supernatant and incubated overnight at 4 °C on a rotating mixer to form antigen–antibody complexes. Gently resuspended Protein A/G magnetic beads with a pipette, transfered 25 µL of the magnetic bead suspension to a 1.5 mL centrifuge tube. Added 500 µL of lysis/wash buffer, gently resuspended the magnetic beads with a pipette, then let the tube sit on a magnetic rack for 1 min. After the beads have adhered to the side of the tube, the supernatant was aspirated. Antigen–antibody complexes were added to the pre‐treated magnetic beads and incubated overnight at 4 °C on a rotating mixer. Let the tube sit on a magnetic rack for 1 min, then aspirated the supernatant after the beads have adhered to the side of the tube. The remaining material in the tube was the antigen–antibody‐magnetic bead complex. Added 500 µL of lysis/wash buffer to the antigen–antibody‐magnetic bead complex obtained in the previous step, gently resuspended the magnetic beads with a pipette, then let the tube sit on a magnetic rack for 1 min. After the beads have adhered to the side of the tube, aspirated the supernatant. Added SDS‐PAGE loading buffer, mixed well, and heated at 100 °C for 10 min. After cooling, let the tube sit on a magnetic rack for 1 min, then collected the supernatant after the beads have adhered to the side of the tube, and proceeded with SDS‐PAGE analysis.

### Molecular Modeling and Docking Analysis

The structures of mouse AKT (PDB ID: 3OW3) were retrieved from the RCSB Protein Data Bank (https://www.rcsb.org/), and obtained the structures of PTK7 (AlphaFold ID: AF‐Q8BKG3‐F1‐v4), POSTN (AlphaFold ID: AF‐Q62009‐F1‐v4) from the AlphaFold Protein Structure Database (https://www.alphafold.ebi.ac.uk/). Then, the Hdock software (http://hdock.phys.hust.edu.cn/) was used to perform docking experiments and generated predicted protein binding modes. Finally, visualized the docking results using PyMOL software (The PyMOL Molecular Graphics System, Version 2.0, Schrödinger, LLC).

### Statistical Analysis

Data were expressed as the mean ± standard deviation (SD) and were analyzed using GraphPad Prism 9.0 (GraphPad Software Inc, USA). For the analysis of quantitative data, normally distributed data were compared between two groups using a two‐tailed Student's *t*‐test and among multiple groups using one‐way analysis of variance (ANOVA). When the assumption of equal variances was met, Bonferroni's post hoc test was applied; in the presence of heteroscedasticity, Tamhane's T2 analysis was used. For data that were not normally distributed, the Mann–Whitney *U*‐test was used for comparisons between two groups, and the Kruskal–Wallis test was used for comparisons among multiple groups. Categorical data were analyzed using the χ^2^‐test. Statistical significance was defined as *p* < 0.05. All experiments were repeated at least three times.

### Ethics

All animal experimental protocols were approved by the Institutional Animal Care and Use Committee (IACUC) of the Shanghai Sixth People's Hospital, and experimental procedures were conducted in strict accordance with the guidelines of the National Institutes of Health Guide for the Care and Use of Laboratory Animals.

## Conflict of Interest

The authors declare no conflict of interest.

## Author Contributions

H.L., X.L., and M.L. contributed equally to this work. H.L. dealt with conceptualization, data curation, investigation, methodology, project administration, software, writing the original draft, and writing the review and editing. X.L. dealt with methodology and writing the original draft. M.L. dealt with data curation and writing the original draft. X.W., Z.S., and J.L. dealt with project administration and writing the original draft. B.X. dealt with project administration and supervision. Q.C., C.F., and H.R. dealt with conceptualization, funding acquisition, investigation, methodology, project administration, supervision, and writing the review and editing. All authors contributed to the content and critical revision and approved the final draft of the manuscript.

## Supporting information



Supporting Information

Supporting Information

## Data Availability

The data that support the findings of this study are available from the corresponding author upon reasonable request.
