# Supplementary material for: POSTN‐Mediated Interplay of M1 Polarized Macrophage with Tendon‐Derived Stem Cells to Drive Traumatic Heterotopic Ossification Formation through PTK7/ATK Signaling?
Source: Adv Sci (Weinh). 2025 Aug 18;12(40):e07951. doi: 10.1002/advs.202507951 (PMC12561399; doi:10.1002/advs.202507951)
Supplement: Supplementary file 1 — Supporting Information [file ADVS-12-e07951-s001.docx]

**
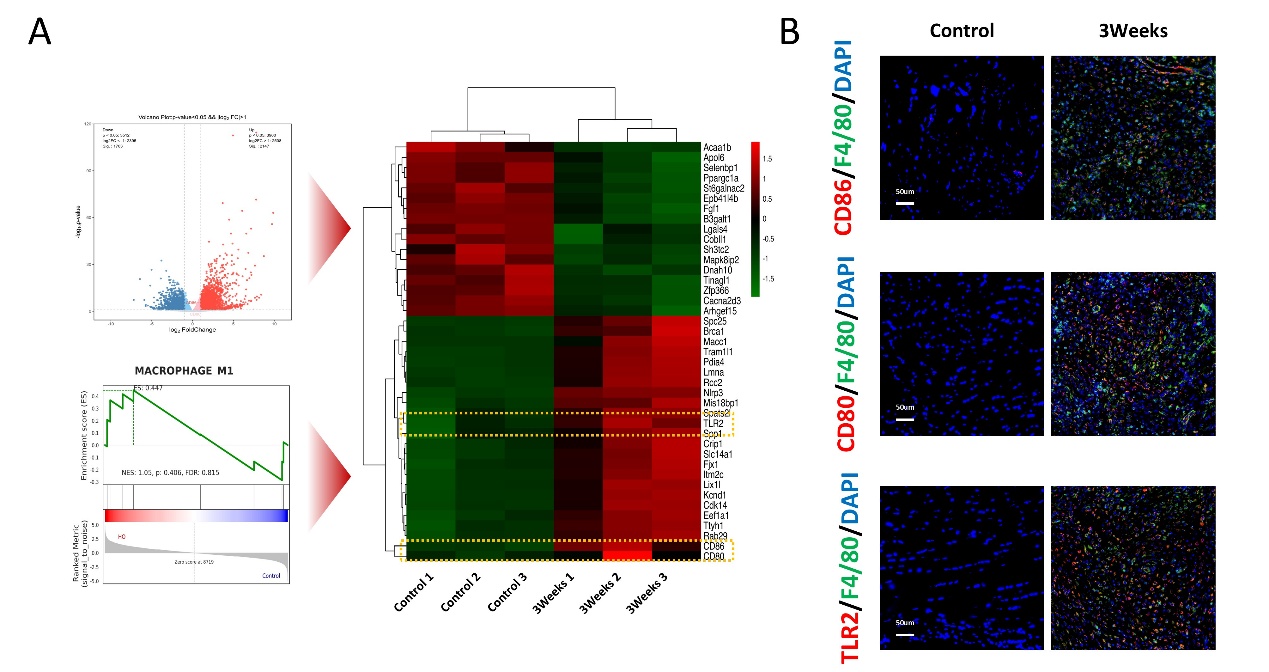
**

**Supplementary Figure 1.**

(A) Gene set enrichment analysis of macrophage M1-associated gene sets, with corresponding volcano plot and heatmap illustrating differential gene expression in Control and HO groups at 3weeks, N=3.

(B) Double immunofluorescence staining for the co-localization of CD86, CD80,TLR2 and F4/80 positive cells in both control and HO groups at 3weeks, N = 3, scale bar = 200 μm.


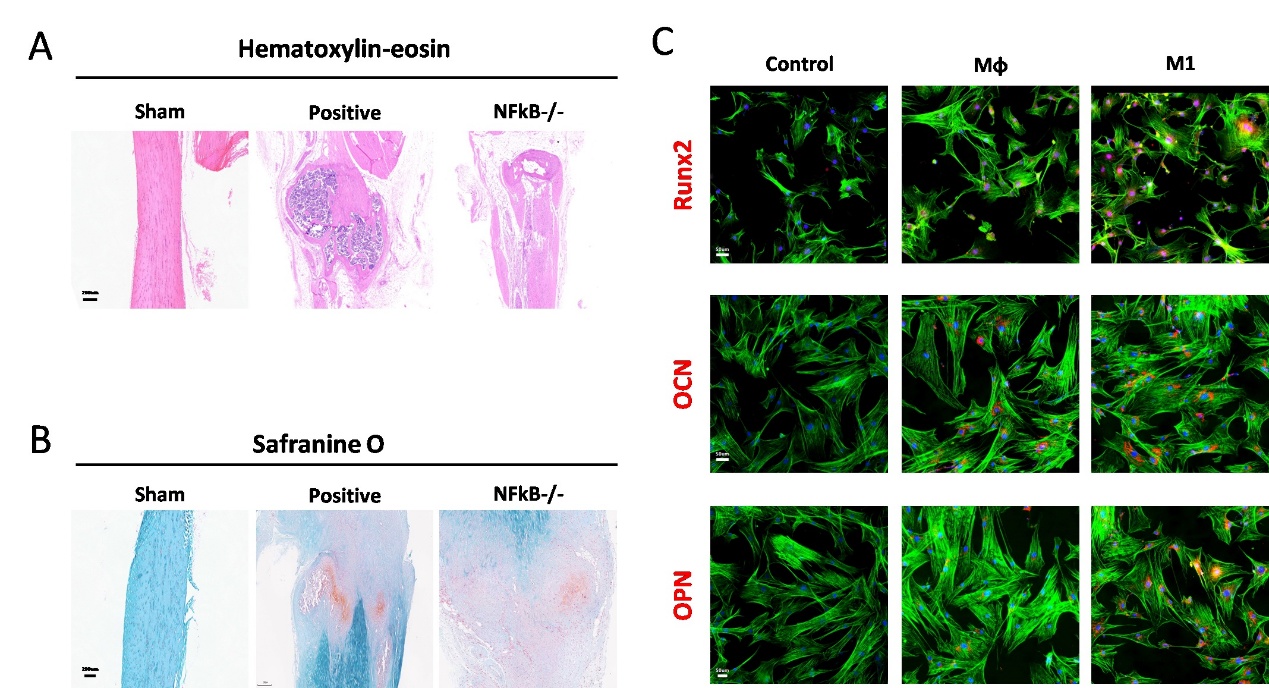


**Supplementary Figure 2.**

(A) H&E staining for tendon in the sham, positive and NFkB-/- groups, N = 6.

(B) Safranine O staining for tendon in the sham, positive and NFkB-/- groups, N = 6.

(C) Immunofluorescence staining for the Runx2, OCN, OPN in the control, Mφ and M1 groups, N = 3


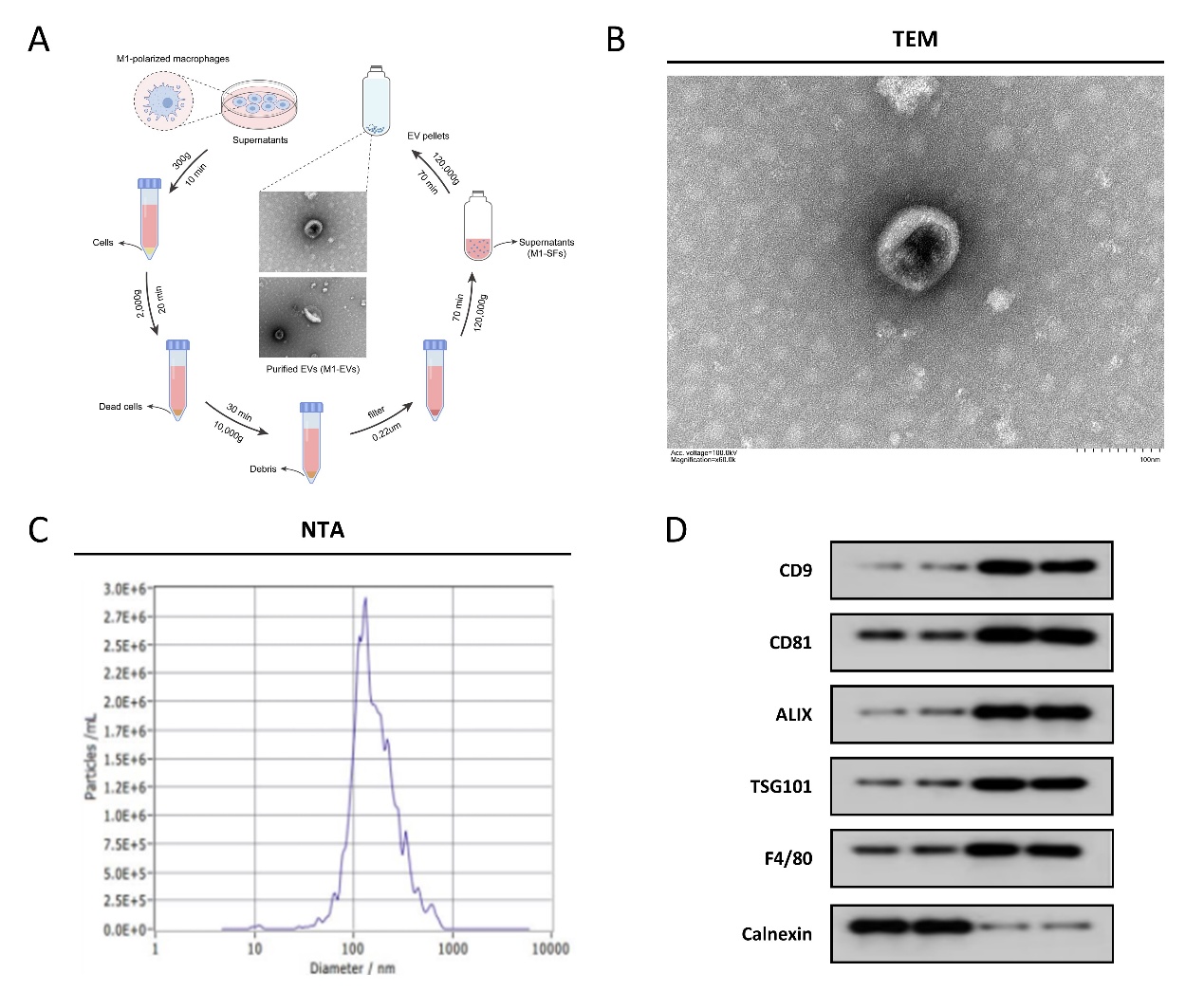


**Supplementary Figure 3**.

(A) Schematic representation of the workflow for the isolation EVs derived from the supernatants of macrophages polarized towards the M0 or M1 phenotype.

(B) Morphological characteristics of EVs by transmission electron microscopic images observation

(C) Nanoparticle tracking analysis (NTA) was used for particle size determination of EVs.

(D)WB analysis was used for identification of the EVs positive markers including CD9, CD81, ALIX, TSG101, negative marker Calnexin and parental cell marker F4/80, N = 3.


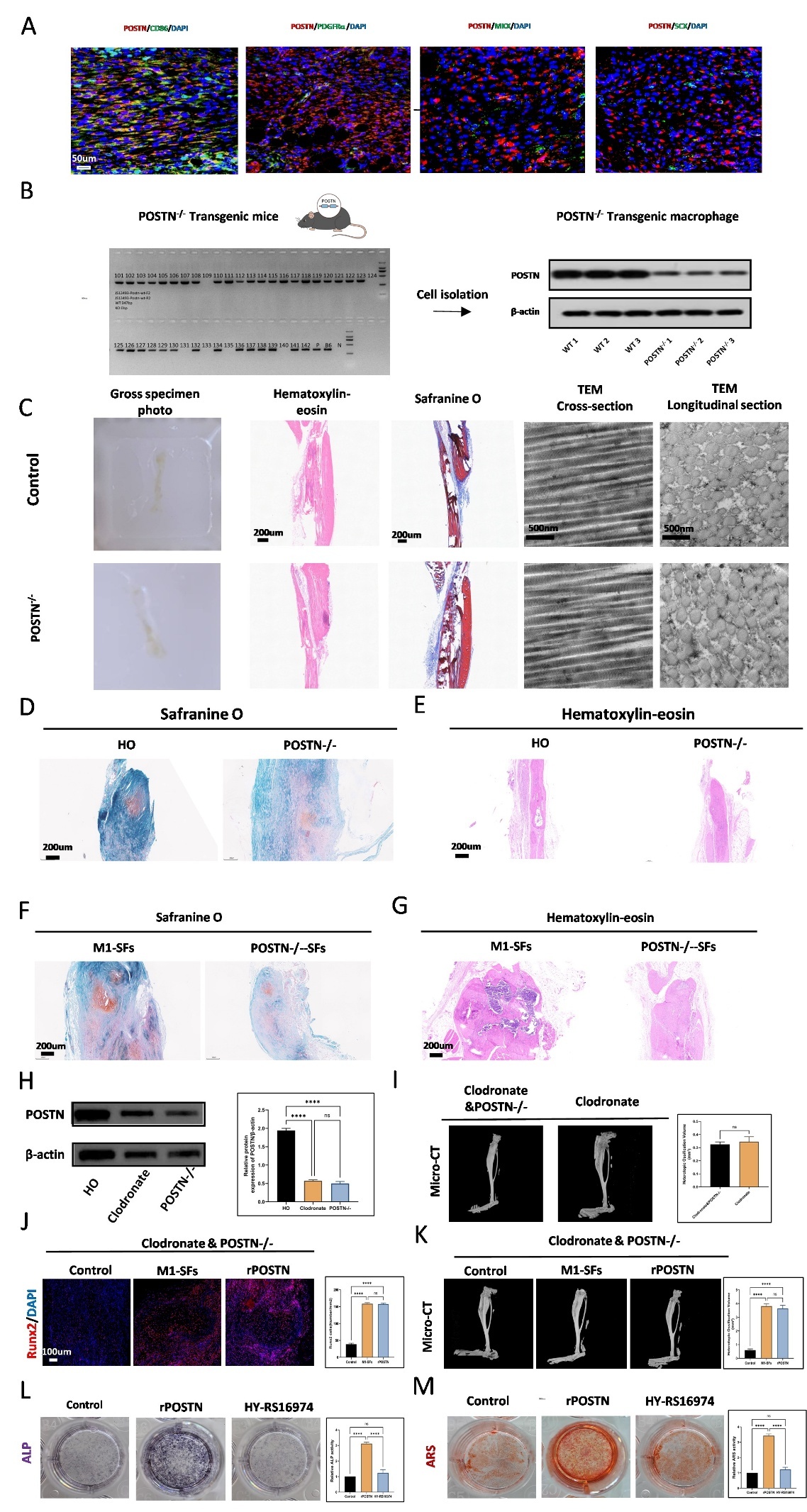


**Supplementary Figure 4.**

1. Immunofluorescence (IF) staining was used to detect the co-localization of POSTN protein with CD86, a marker of M1 macrophages, and classical TDSC markers (PDGFRα, MKX and SCX) , N=6.
2. Schematic representation of transgenic mice with POSTN knockout, along with glue-running maps and WB validation of POSTN protein reduction, N = 6.
3. Macroscopic observations of tendon morphology, histological analysis (H&E and Masson's trichrome staining), and ultrastructural evaluation of collagen architecture via transmission electron microscopy (TEM) were used to assess tendon homeostasis in POSTN knockout mice and wild-type mice.
4. Safranine O staining was performed on the tendons of the HO group and the group with POSTN knockout, N = 6.
5. H&E staining was performed on the tendons of the HO group and the group with POSTN knockout, N = 6.
6. Safranine O staining for tendon in the M1-SFs and M1-SFs with POSTN knockout groups, N = 6.
7. H&E staining for tendon in the M1-SFs and M1-SFs with POSTN knockout groups, N = 6
8. WB was used to detect the expression levels of POSTN protein in the HO group, the group with macrophages cleared by clodronate, and the POSTN knockout group, N =3, **** p < 0.0001.
9. Micro-CT was used to detect the bone mass of heterotopic ossification in the group where POSTN-knockout mice were treated with clodronate and the group where clodronate was used alone, N = 6, p ＞ 0.05.
10. Runx2 immunofluorescence staining in mice treated with clodronate and rPOSTN protein respectively, including the group of mice with clodronate treatment and the group of POSTN - knockout mice., N = 6, **** p < 0.0001
11. Micro-CT was used to detect the bone mass of heterotopic ossification in mice treated with clodronate and rPOSTN protein respectively, including the group of mice with clodronate treatment and the group of POSTN - knockout mice., N = 6, **** p < 0.0001.
12. ALP staining were used to detect the osteogenesis of TDSCs in the Control, rPOSTN and HY-RS16974 groups, N =6, **** p < 0.0001.
13. ARS staining were used to detect the osteogenesis of TDSCs in the Control, rPOSTN and HY-RS16974 groups, N =6, **** p < 0.0001.


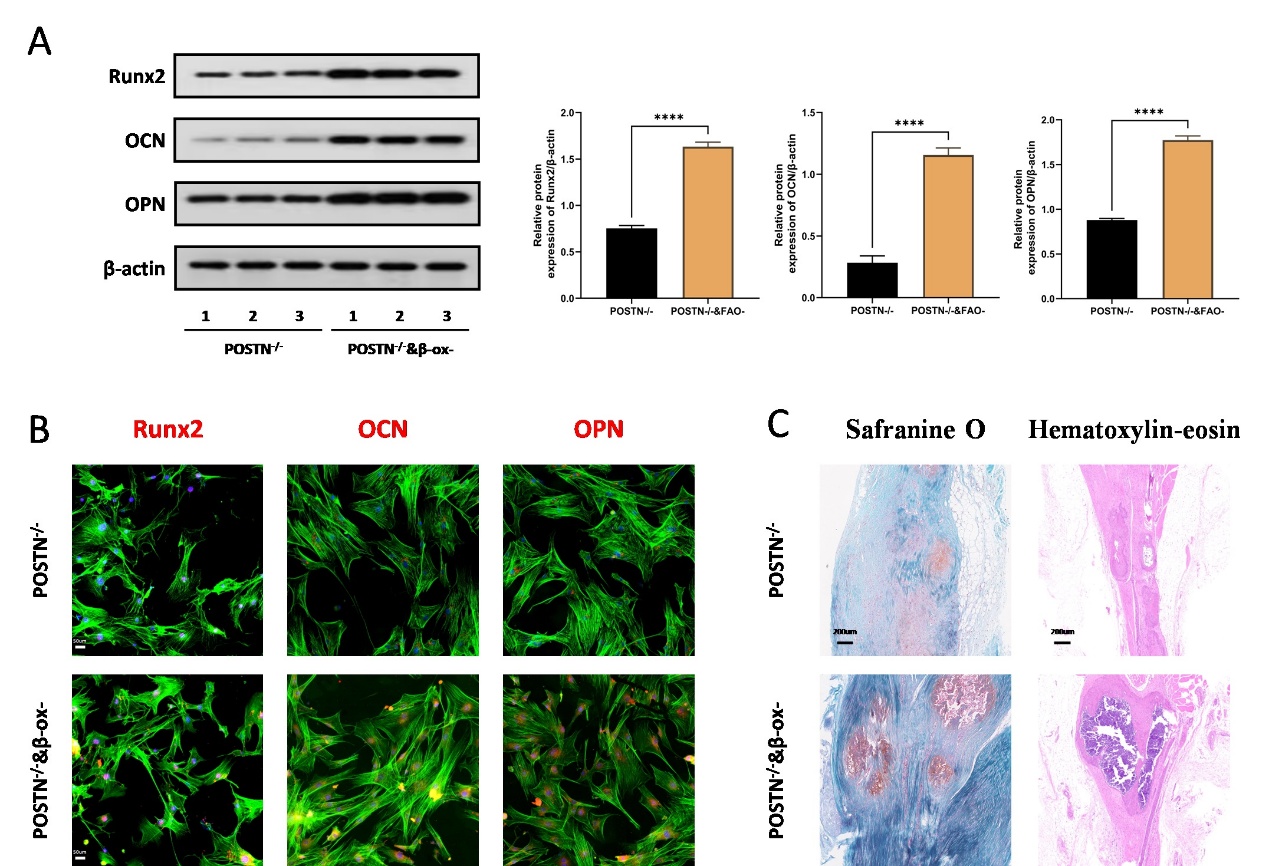


**Supplementary Figure 5.**

1. WB analysis was used to detect the expression of osteogenic related protein levels (Runx2, OCN, OPN) for TDSCs in addition of M1-SFs with POSTN knockout groups, with or without sh-LCAD, N = 3, **** p < 0.0001.
2. Immunofluorescence staining was used to detect the expression of osteogenic related protein levels (Runx2, OCN, OPN) for TDSCs in addition of M1-SFs with POSTN knockout groups, with or without sh-LCAD, N = 3.
3. Safranine O and H&E staining for tendon in the M1-SFs with POSTN knockout groups, with or without sh-LCAD, N = 6.


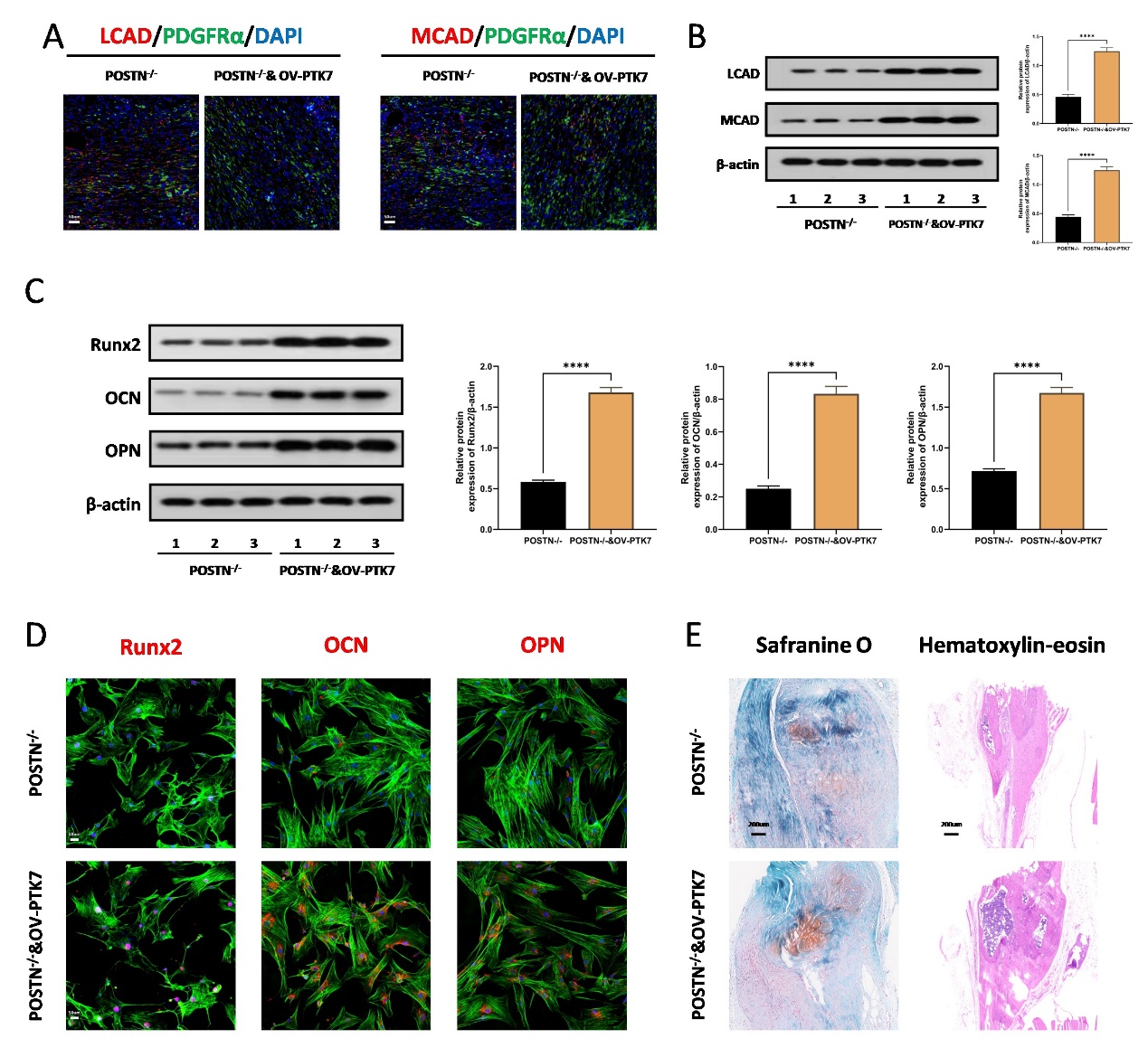


**Supplementary Figure 6.**

1. WB analysis was used to detect the expression of osteogenic related protein levels (Runx2, OCN, OPN) for TDSCs in the POSTN-/- and POSTN-/-&OV-PTK7 groups, N=3, **** p < 0.0001.
2. Immunofluorescence staining was used to detect the expression of osteogenic related protein levels (Runx2, OCN, OPN) for TDSCs in the POSTN-/- and POSTN-/-&OV-PTK7 groups, N = 3.
3. Safranine O and H&E staining for tendon in the POSTN-/- and POSTN-/-&OV-PTK7 groups, N = 6.

**
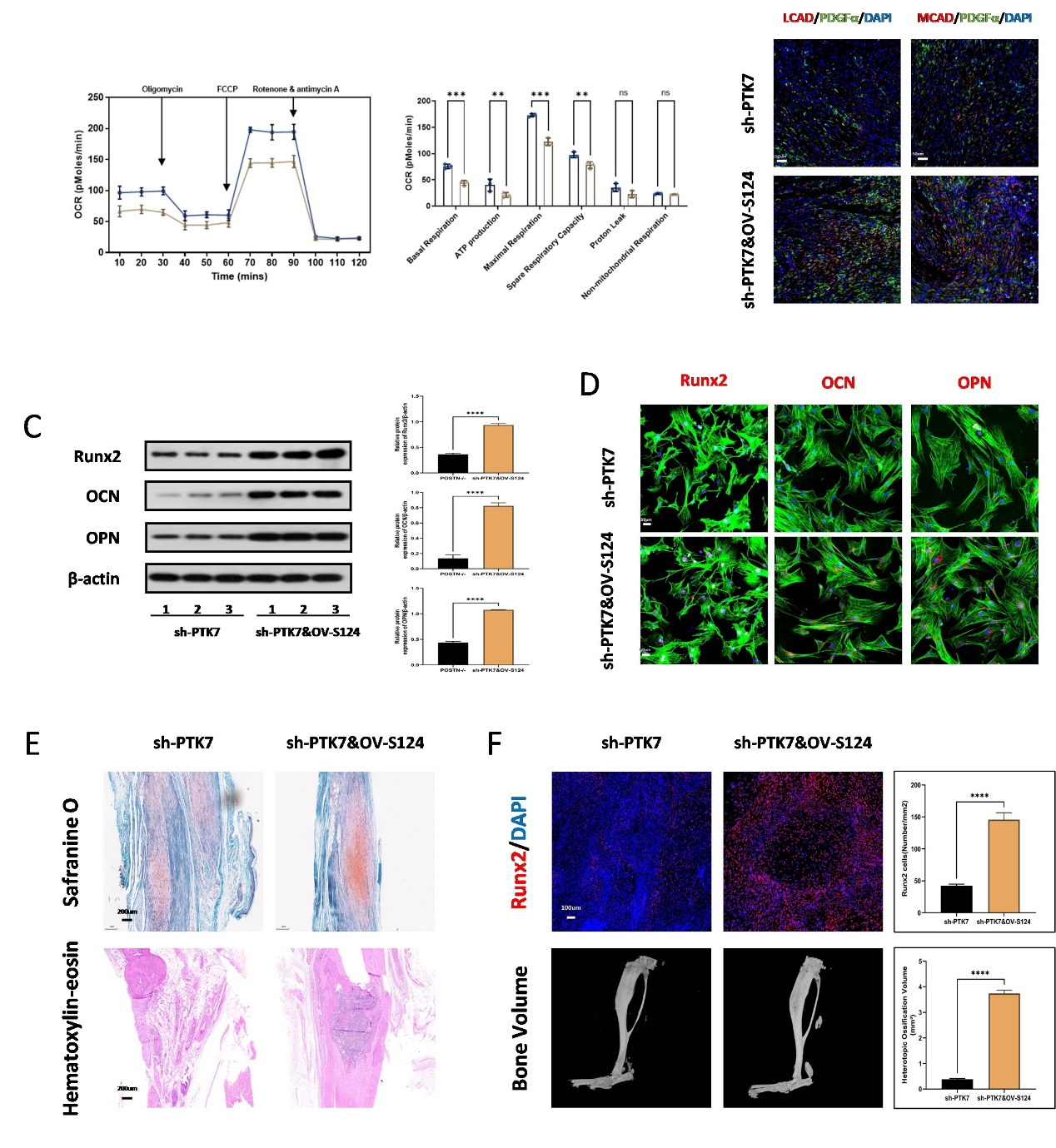
**

**Supplementary Figure 7**.

1. Seahorse test was used to detect the oxidative phosphorylation level in the osteogenic induced TDSCs in addition of PTK7 knockout with or without the serine 124 - site overexpression of AKT groups, N = 3, ● represented PTK7 knockout groups and ▲ represented PTK7 knockout with the serine 124 - site overexpression of AKT groups.
2. Immunofluorescence staining for the LCAD and MCAD (red), co-localized with PDGFRα(green) of tendons in addition of PTK7 knockout with the serine 124 - site mutation of AKT groups, N =3.
3. WB analysis was used to detect the expression of osteogenic related protein levels (Runx2, OCN, OPN) for TDSCs in the sh-PTK7 and sh-PTK7&OV-S124 groups, N=3, **** p < 0.0001.
4. Immunofluorescence staining was used to detect the expression of osteogenic related protein levels (Runx2, OCN, OPN) for TDSCs in the sh-PTK7 and sh-PTK7&OV-S124 groups, N = 3.
5. Safranine O and H&E staining for tendon in the sh-PTK7 and sh-PTK7&OV-S124 groups, N = 6.
6. Immunofluorescence staining for the Runx2 of tendons and Micro-CT analysis of HO formation in the sh-PTK7 and sh-PTK7&OV-S124 groups, N =6, **** p < 0.0001.


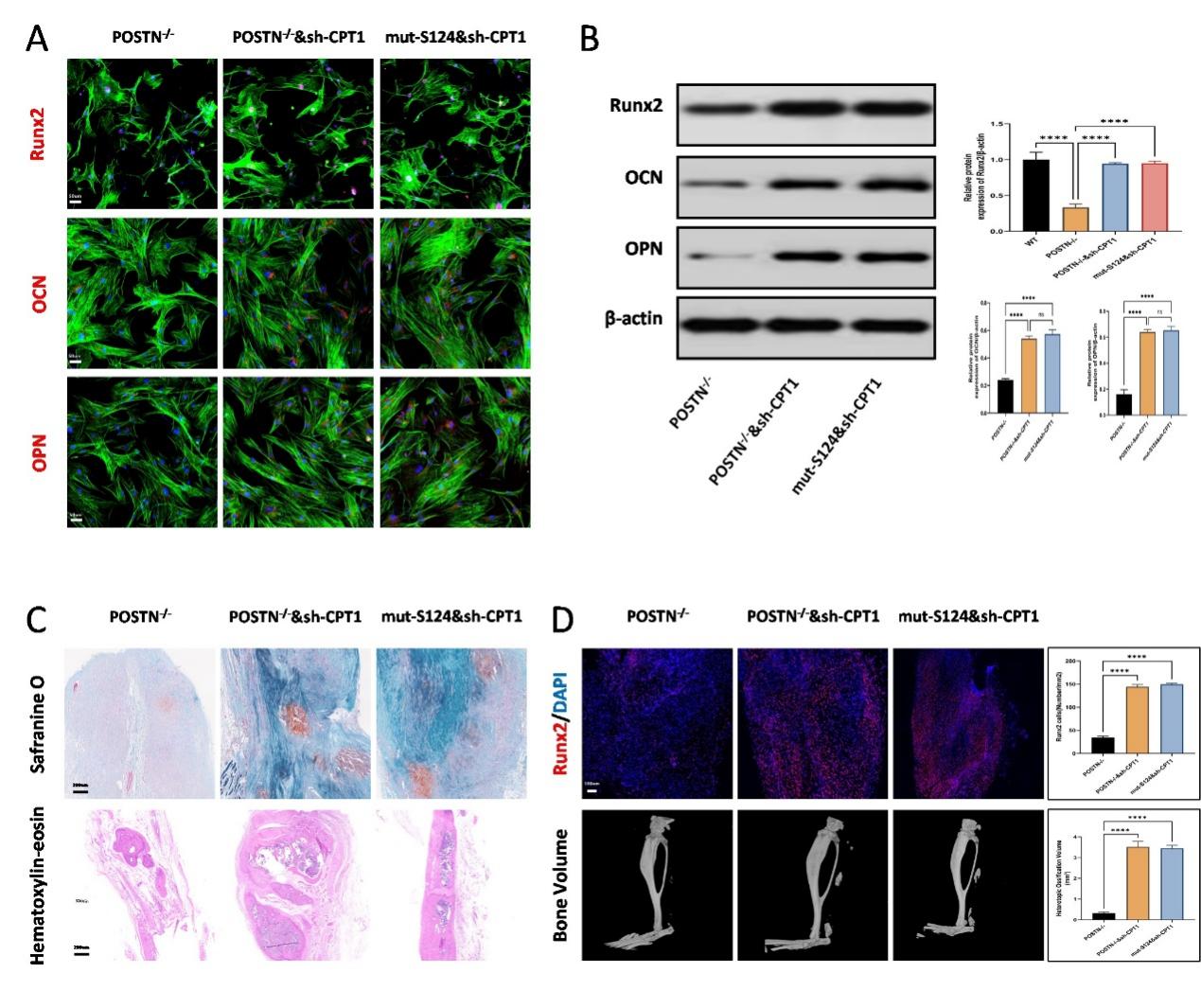


**Supplementary Figure 8**.

1. Immunofluorescence staining was used to detect the expression of osteogenic related protein levels (Runx2, OCN, OPN) for TDSCs in the POSTN-/-, POSTN-/-&sh-CPT1 and mut-S124&sh-CPT1 groups, N = 3.
2. WB analysis was used to detect the expression of osteogenic related protein levels (Runx2, OCN, OPN) for TDSCs in the POSTN-/-, POSTN-/-&sh-CPT1 and mut-S124&sh-CPT1 groups, N=3, **** p < 0.0001
3. Safranine O and H&E staining for tendon in the POSTN-/-, POSTN-/-&sh-CPT1 and mut-S124&sh-CPT1 groups, N = 6.
4. Immunofluorescence staining for the Runx2 of tendons and micro-CT analysis of HO formation in the POSTN-/-, POSTN-/-&sh-CPT1 and mut-S124&sh-CPT1 groups, N =6, **** p < 0.0001.


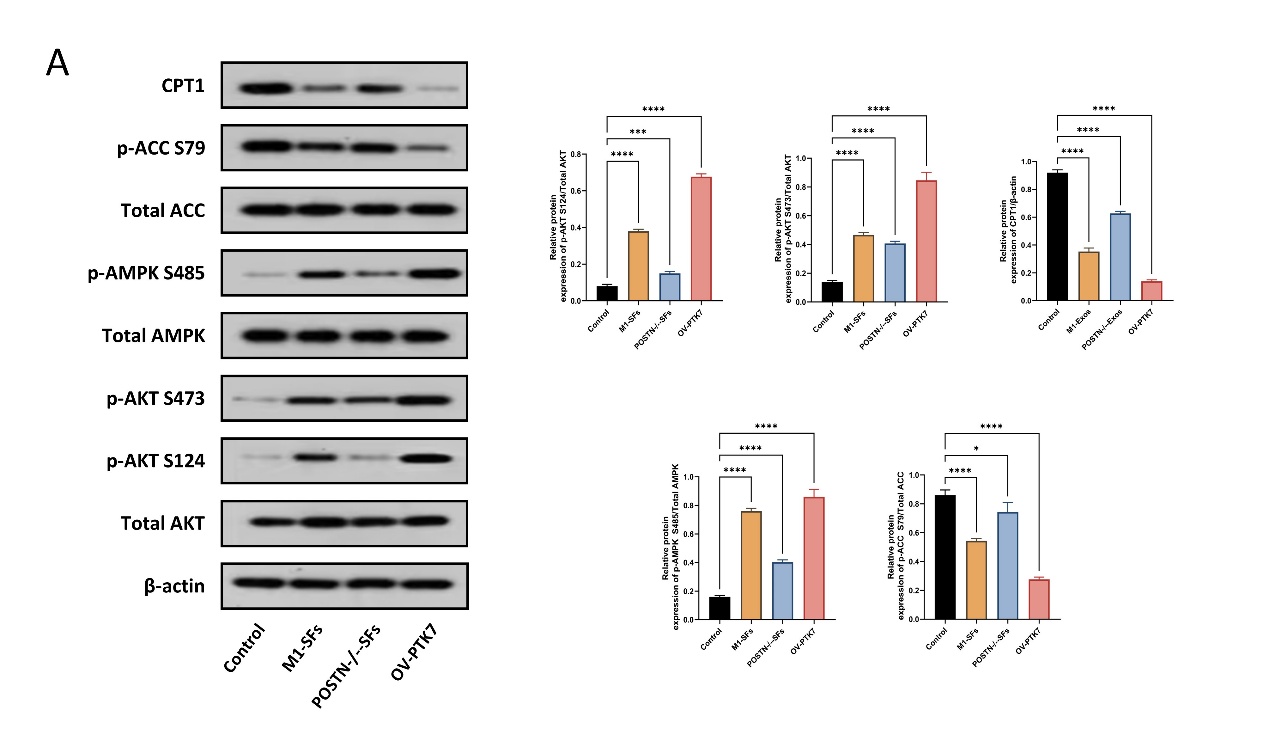


**Supplementary Figure 9**.

1. WB analysis was used to detect the expression of CPT1, p-ACC S79, ACC, p-AMPK S485, AMPK, p-AKT S473, p-AKT S124 and AKT protein levels for TDSCs in the control, M1-SFs, POSTN-/--SFs and OV-PTK7 groups, N=3, * p < 0.1, *** p < 0.001, **** p < 0.0001.


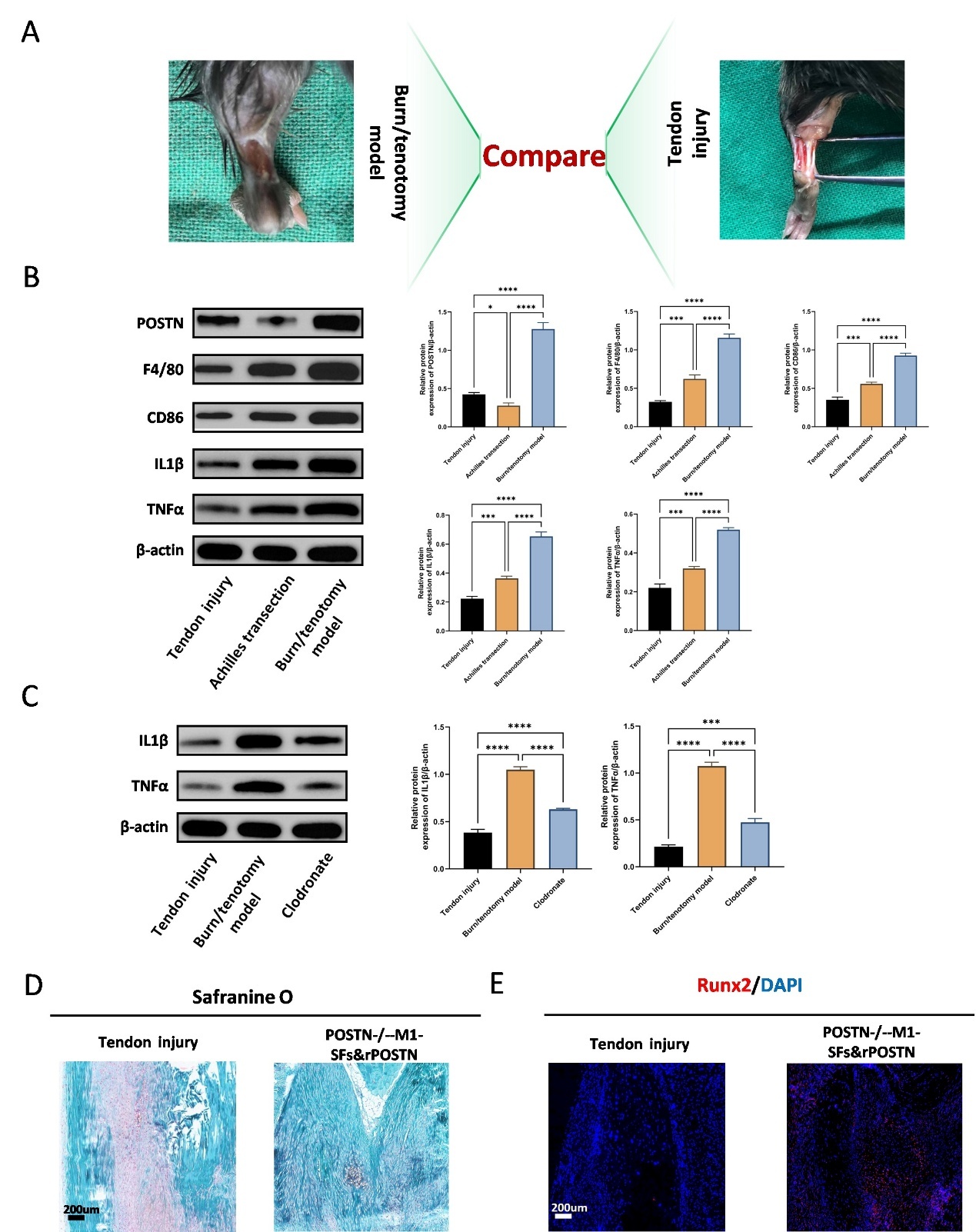


**Supplementary Figure 10**.

1. Schematic diagrams of Achilles tendon modeling using the Burn/tenotomy model and tendon injury models.
2. WB analysis was used to detect the expression of POSTN, F4/80, CD86, IL1β and TNFα protein levels in the tendon injury, Achilles transection , and burn/tenotomy model groups, N=3, * p < 0.1, *** p < 0.001, **** p < 0.0001.
3. WB analysis was used to detect the expression of IL1β and TNFα protein levels in the tendon injury, burn/tenotomy model and clodronate groups, N=3, * p < 0.1, *** p < 0.001, **** p < 0.0001.
4. Safranine O-fast green staining for tendon in the tendon injury and POSTN-/--M1-SFs&rPOSTN groups, N = 6.
5. Immunofluorescence staining for the Runx2 of tendons in the tendon injury and POSTN-/--M1-SFs&rPOSTN groups, N = 6.
