# Supplementary material for: POSTN‐Mediated Interplay of M1 Polarized Macrophage with Tendon‐Derived Stem Cells to Drive Traumatic Heterotopic Ossification Formation through PTK7/ATK Signaling?
Source: Adv Sci (Weinh). 2025 Aug 18;12(40):e07951. doi: 10.1002/advs.202507951 (PMC12561399; doi:10.1002/advs.202507951)
Supplement: Supplementary file 2 — Supporting Information [file ADVS-12-e07951-s002.docx]

**Supplementary Table S1.** Primers used for genotyping the NF-κB-/- mice.

| PCR No. | Primer No. | Sequence | Band Size |
| --- | --- | --- | --- |
| PCR① | T007133-F1 | GAGGCATCAACAGGGCTAC | WT:244bp  Targeted: 325bp |
|  | T007133-R1 | CATGTGCCATCAATGTAGGAC |  |
| PCR② | T007133-F1 | GGGGAAACAGTAGGAAAGTAAC | WT:252bp  Targeted: 330bp |
|  | T007133-R2 | GCATCTCCACCAACTAACAAG |  |

**Supplementary Table S1.** Primers used for genotyping the POSTN-/- mice.

| PCR No. | Primer No. | Sequence | Band Size |
| --- | --- | --- | --- |
| PCR① | T011526-F1 | AAATGCACAGAGCCCTGGGTTT | WT:1502bp  KO:257bp |
|  | T011526-R1 | TTAACCAAGCACTGGGTCTCAGC |  |
| PCR② | T011526-F2 | CATGGGAGCTTTTCCTCTGTGA | WT:347bp  KO:0bp |
|  | T011526-R2 | CCGTTACACATTCAGAGCTGGTGAG |  |
